# Supplementary material for: Immune correlates of HIV-1 reservoir cell decline in early-treated infants
Source: Cell Rep. 2022 Jul 19;40(3):111126. doi: 10.1016/j.celrep.2022.111126 (PMC9314543; doi:10.1016/j.celrep.2022.111126)
Supplement: Document S1. Figures S1–S5 and Tables S1 and S2 [file mmc1.pdf]

Cell Reports, Volume *40*

Supplemental information

Immune correlates of HIV-1 reservoir cell decline in early-treated infants

Ciputra Adijaya Hartana, Pilar Garcia-Broncano, Yelizaveta Rassadkina, Xiaodong Lian, Chenyang Jiang, Kevin B. Einkauf, Kenneth Maswabi, Gbolahan Ajibola, Sikhulile Moyo, Terence Mohammed, Comfort Maphorisa, Joseph Makhema, Yuko Yuki, Maureen Martin, Kara Bennett, Patrick Jean-Philippe, Mathias Viard, Michael D. Hughes, Kathleen M. Powis, Mary Carrington, Shahin Lockman, Ce Gao, Xu G. Yu, Daniel R. Kuritzkes, Roger Shapiro, and Mathias Lichterfeld

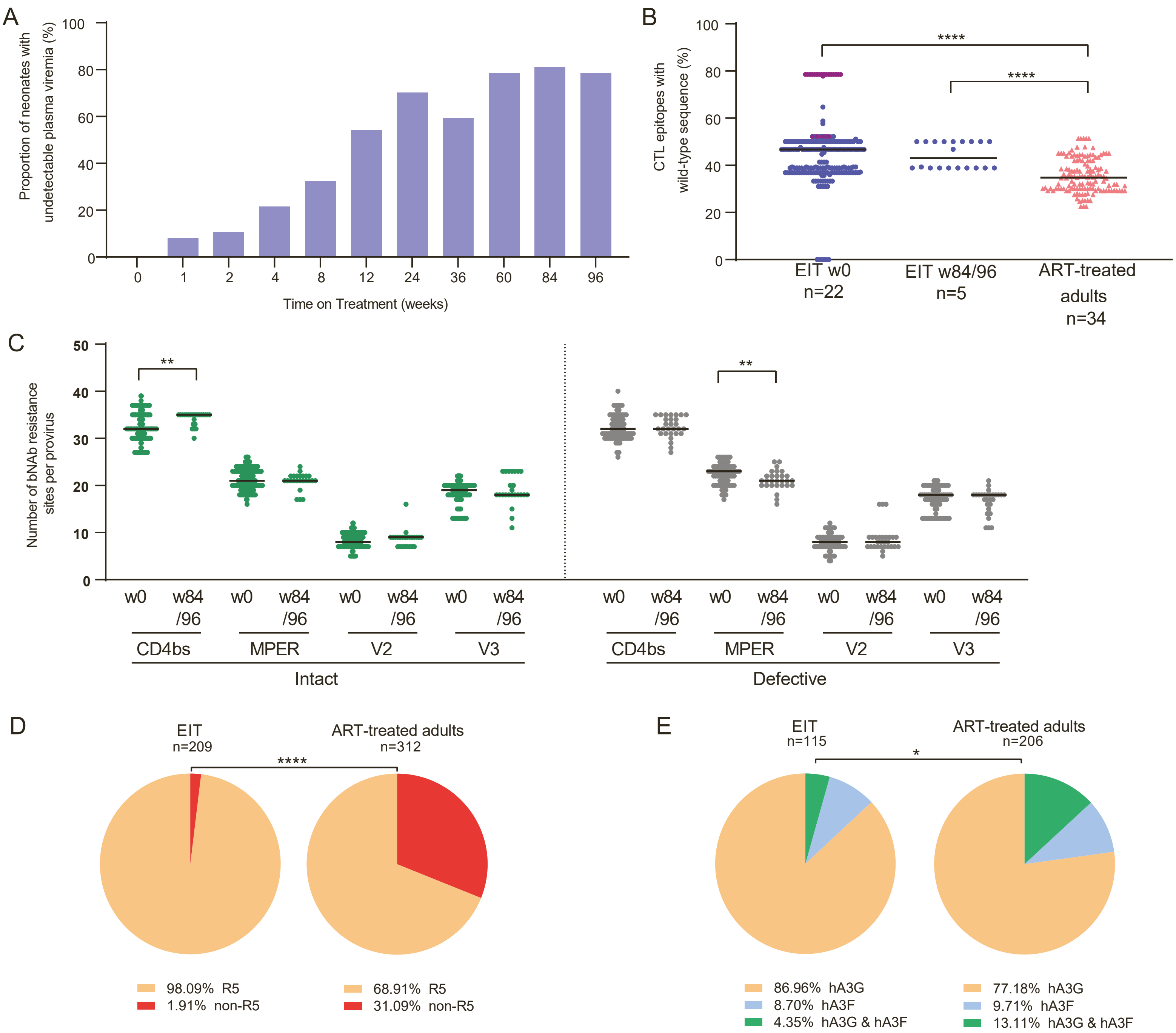

Supplemental Figure 1: Proviral reservoir features in HIV-1-infected neonates. (A): Proportion of neonates in the EIT study with undetectable plasma viremia at indicated timepoints after birth. (B): Proportion of CTL epitopes (restricted by autologous HLA class I alleles) within intact proviruses that display the clade C wild-type sequence (for clade C infected infants from Botswana at week 0 and week 84/96 after early ART) or the clade B wild-type sequences (for clade B-infected adults from the US). ****p<0.0001; Kruskal Wallis test with post-hoc Dunn's test. Data from infants with peripartal infection are indicated in purple. (C): Numbers of amino acid residues associated with resistance to broadly-neutralizing antibodies, determined as described before (Bricault et al., 2019), in intact proviruses from EIT study participants. Data for four classes of bnAbs [CD4 binding site (CD4bs), membrane proximal external region (MPER), V2 domain and V3 domain] at week 0 and week 84/96 are shown. **p<0.01, Mann Whitney U test adjusted for multiple testing. (D): Proportion of R5-tropic and non-R5-tropic intact proviruses from early-treated infants, relative to ART-treated adults. R5 tropism was determined using the Geno to Pheno algorithm with a proportional FPR ≥ 5.75%. ****p<0.0001; Fisher's exact test. (E): Proportion of near-full proviral sequences with footprints of hypermutations mediated by APOBEC3G, APOBEC3F or both. *p<0.05; Chi square test.

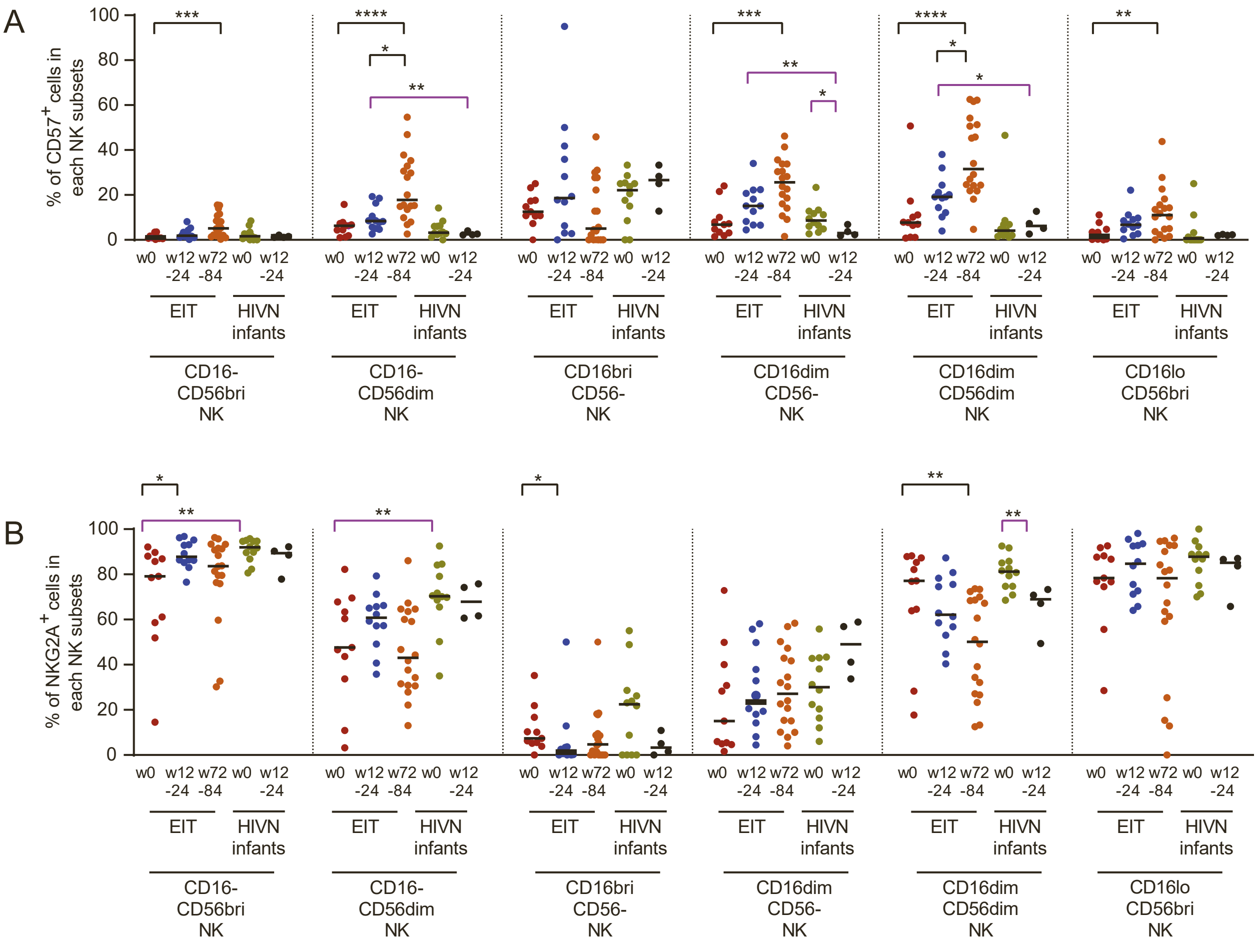

Supplemental Figure 2: NK cell responses of infants receiving early ART compared to HIV-negative infants. (A-B): Longitudinal evolution of CD57-expressing (A) and NKG2A-expressing (B) NK cell subsets in early-treated infants. Data from week 0, week 12/24 and week 72/84 are shown in comparison to HIV-negative infants at week 0 and week 12-24. *p<0.05, **p<0.01, ***p<0.001, ****p<0.0001; Kruskal Wallis test with post-hoc Dunn's test to compare longitudinal data from EIT infants (black line) or Mann Whitney U test adjusted for multiple testing to compare data between similar timepoints from EIT and HIV-negative infants (purple line).

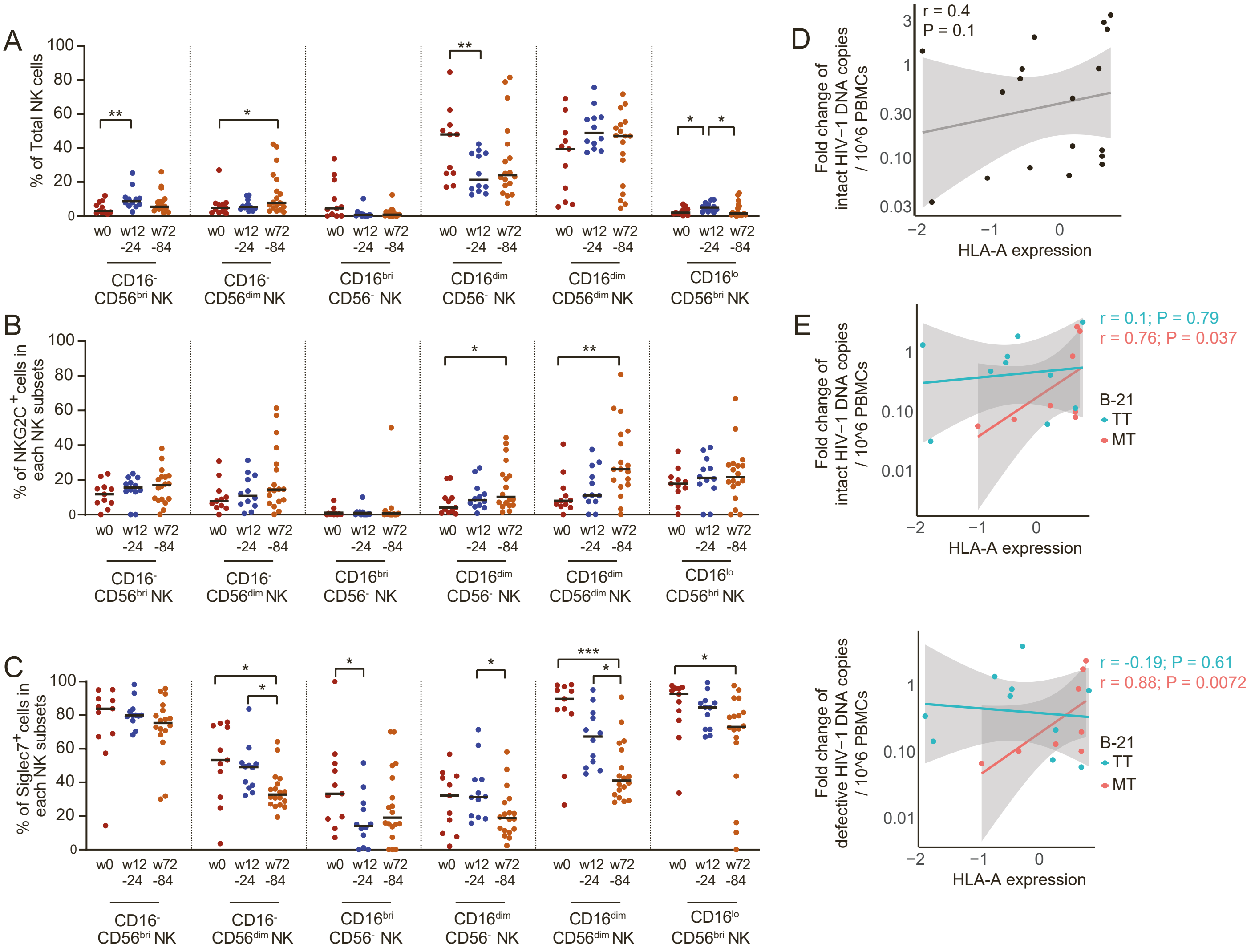

Supplemental Figure 3: Associations between NK cell responses and intact proviral reservoir sequences in early-treated infants. (A): Proportions of indicated NK cell subsets defined by CD16 and CD56 expression in early-treated infants at indicated timepoints. (B-C): Proportions of indicated NK cell subsets expressing NKG2C (B) or Siglec-7 (C) at indicated timepoints. *p<0.05, **p<0.01, ***p<0.001; Kruskal Wallis test with post-hoc Dunn's test. (D): Association between fold-change of intact proviruses between week 0 and week 72/84 and corresponding levels of HLA-A surface expression, determined as described before (Ramsuran et al., 2018). (E): Associations between fold-change of intact (top) and defective (bottom) proviruses between week 0 and week 72/84 and levels of of HLA-A expression. Participants were stratified according to presence or absence of the -21TT or MT genotype. (D-E): Spearman association coefficients are shown.

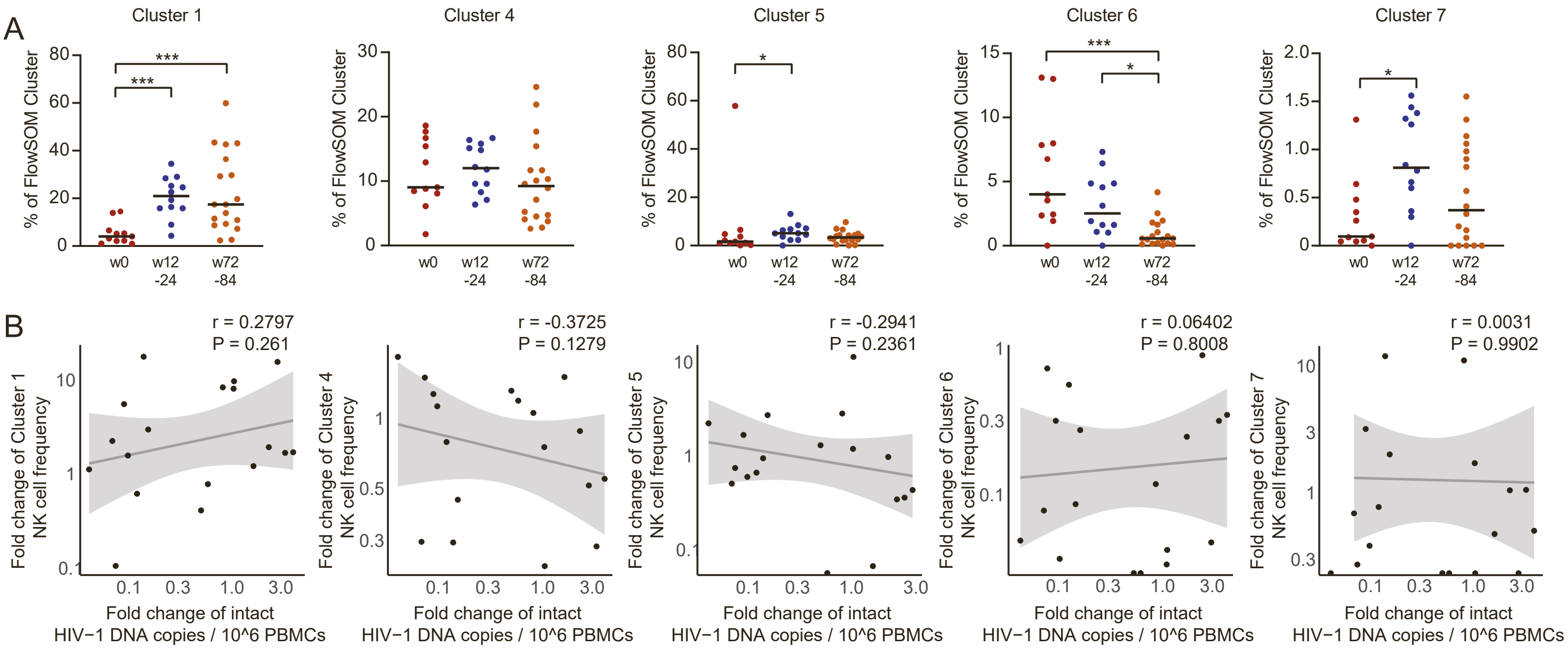

Supplemental Figure 4: Longitudinal associations between NK cell subgroups and intact proviral reservoir sequences in early-treated infants. (A): Longitudinal evolution of NK cell clusters defined through a computational data analysis approach using FlowSOM within the CD56^{dim} CD16^{dim} NK cell compartment, as shown in Figure 3B. (B): Associations between longitudinal changes (between week 0 and week 72/84) of indicated NK cell clusters identified in Figure 3B and corresponding frequencies of intact proviruses. Spearman correlation coefficient is shown. *p<0.05, **p<0.01, ***p<0.001; Kruskal Wallis test with post-hoc Dunn's test.

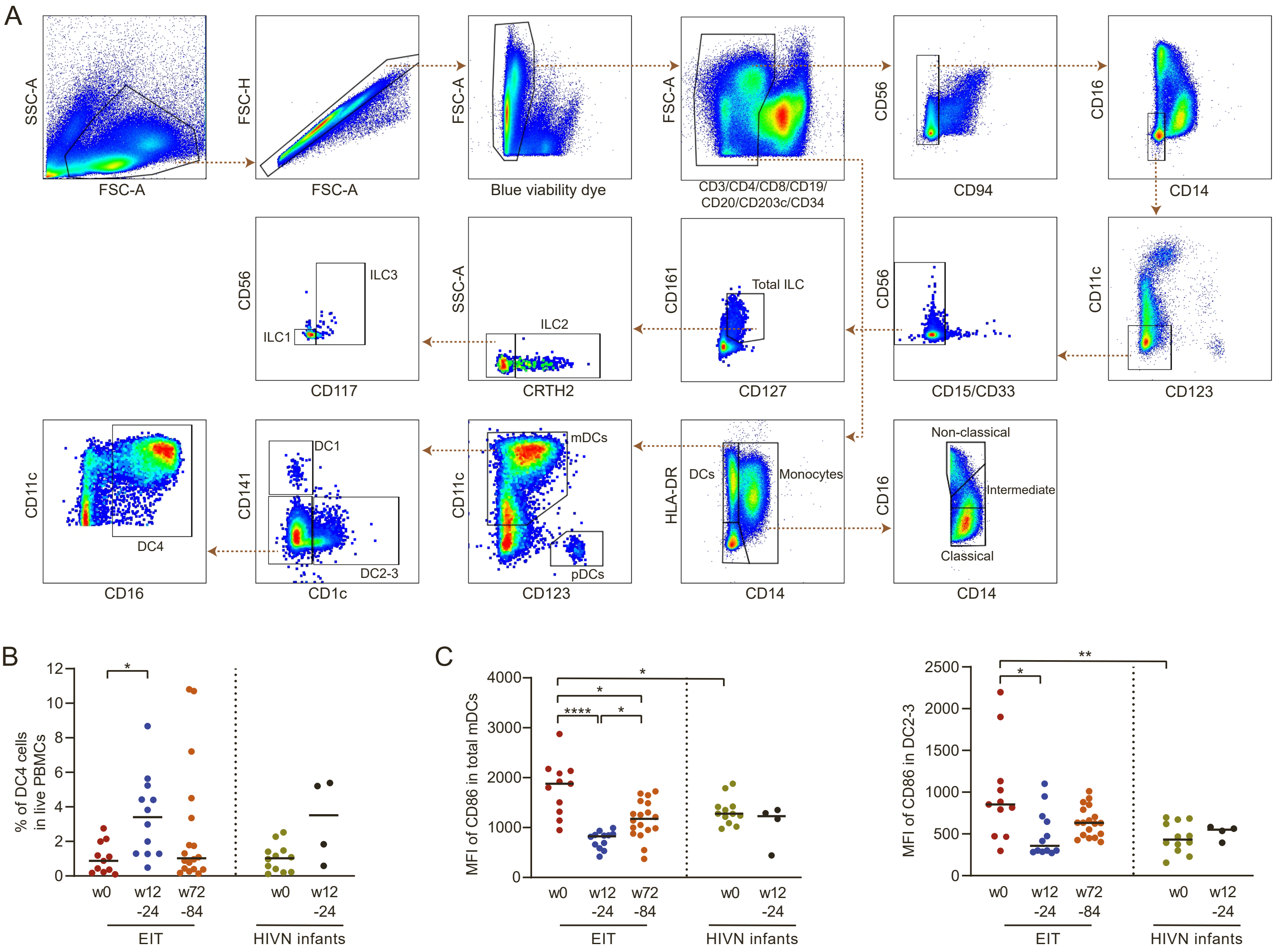

Supplemental Figure 5: Flow cytometric analysis of innate lymphoid cells. (A): Flow cytometric gating strategy for analyzing innate lymphoid cells (ILCs), myeloid dendritic cells (mDCs), plasmacytoid dendritic cells (pDCs) and monocytes. (B): Proportion of DC4 within all PBMC at indicated timepoints in early-treated HIV-1-infected infants (EIT) and in HIV negative infants. (C): MFI of CD86 in total mDCs (left) and DC2-3 (right). *p<0.05, **p<0.01, ****p<0.0001; Kruskal Wallis test with post-hoc Dunn’s test and Mann Whitney U test adjusted for multiple testing.

Supplementary Table 1: Clinical and demographical characteristics of study cohort.

Characteristic	EIT n=37	Control infant with later ART initiation n=10	HAART n=41	HIV-1 negative infants n=16
**Age (years)***	As indicated in text	2.2 ± 0.2	53.5 ± 8.6	As indicated in text
**Recruitment site**	Botswana	Botswana	Boston	Botswana
**Time (days) between HIV-1 infection and ART initiation***	mean of 1 day	161.4 ± 95	ND	NA
**Time (years) on ART***	As indicated in text	1.7 ± 0.3	13.1 ± 5.3	NA
**Viral Load (copies/mL)***	887,250.6 ± 2,751,392#	<40	<40	NA

*Values are express as mean ± SD

Viral load at enrollment

ND = Not Determined

NA = Not Applicable

Supplementary Table 2: Flow cytometry antibodies used for immune cell phenotyping.

Panel 1: NK/ILC/DC/Monocytes cells					
	**Marker**	**Fluorochrome**	**Clone**	**Reference**	**Vendor**
1	CD94	BB790	HP-3D9	624296	BD Bioscience
2	Lin 1*	BB700	-	*see below	BD Bioscience
3	CD123	BB660	7G3	624295	BD Bioscience
4	CD141	BB630	1A4	624294	BD Bioscience
5	NKG2A	VioBright FITC	REA110	130-113-568	Miltenyi
6	CD57	PE-Cy7	HNK-1	359624	BioLegend
7	CD11c	PE-Cy5.5	BU15	MHCD11C18	Invitrogen
8	CD294 (CHTR2)	PE-dazzle 594	BM16	350126	BioLegend
9	Siglec7	PE	G-434	339204	BioLegend
10	CD127	APC-Fire750	A019D5	351350	BioLegend
11	CD64	AF700	10.1	305040	BioLegend
12	CD158e1/e2	APC	REA168	130-104-485	Miltenyi
13	CD117	BV750	104D2	747514	BD Bioscience
14	NKp30 (CD337)	BV711	P30-15	563383	BD Bioscience
15	CD161	BV650	DX12	563864	BD Bioscience
16	Lin2٢	Biotin	-	٢See below	BioLegend
	streptavidin	BV570	-	405227	BioLegend
17	CD16	BV480	3G18	566108	BD Bioscience
18	NKG2C	BV421	134591	748169	BD Bioscience
19	CD14	BUV805	35E2	612902	BD Bioscience
20	CD56	BUV737	NCAM16.2	564447	BD Bioscience
21	HLADR	BUV661	G46-6	565073	BD Bioscience
22	CD86	BUV563	2331FUN11	741386	BD Bioscience
23	CD1c	BUV395	F10/21A3	742751	BD Bioscience

*CD3/CD4/CD8/CD19/CD20/CD203c/CD34_**566575** (SK7) /**566452** (RPAT8) /**566392** (SK3) /**566396** (SJ25C1) /**745889** (2H7) /**745913** (NP4D6) /**742246** (563)

٢CD15/CD33_**301914** (HI98) /**303426** (W1453)
