# Supplementary material for: Immune correlates of HIV-1 reservoir cell decline in early-treated infants
Source: Cell Rep. 2022 Jul 19;40(3):111126. doi: 10.1016/j.celrep.2022.111126 (PMC9314543; doi:10.1016/j.celrep.2022.111126)
Supplement: Document S2. Article plus supplemental information [file mmc2.pdf]

Cell Reports

Report

Immune correlates of HIV-1 reservoir cell decline in early-treated infants

Graphical abstract

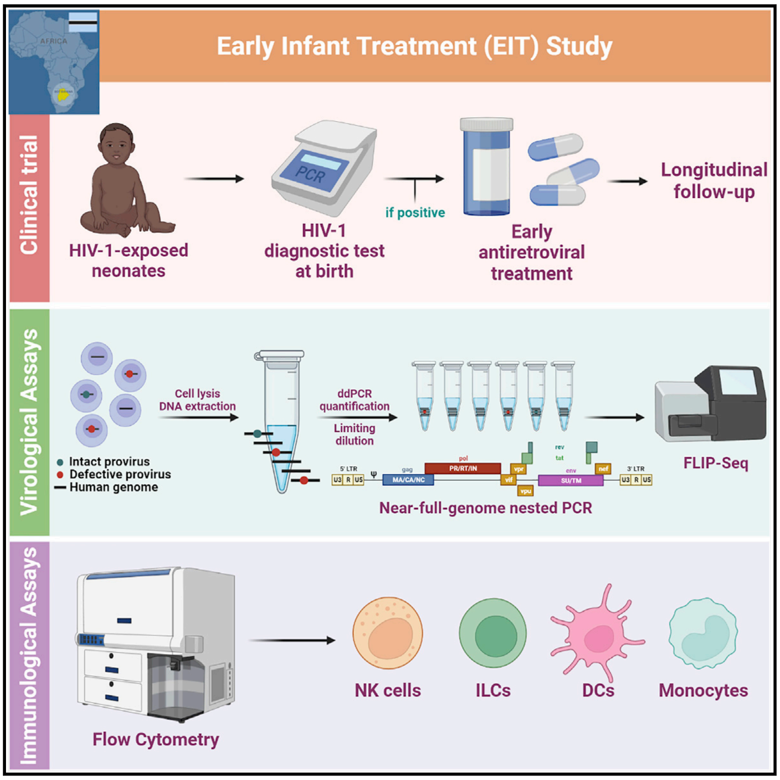

Highlights

- ART initiation in neonates leads to a profound decline of intact HIV-1 proviruses
- The longitudinal decline of intact HIV-1 proviruses is linked to NK cell responses
- Initiation of ART in neonates corrects innate immune perturbations in ILCs and DCs
- ILCs, macrophages, and DCs are not associated with a decline of intact HIV-1 proviruses

Authors

Ciputra Adijaya Hartana, Pilar Garcia-Broncano, Yelizaveta Rassadkina, ..., Daniel R. Kuritzkes, Roger Shapiro, Mathias Lichterfeld

Correspondence

mlichterfeld@partners.org

In brief

Hartana et al. report longitudinal assessments of HIV-1 reservoir cells and innate immune responses in 37 infected neonates from Botswana who started antiretroviral treatment (ART) early, frequently within hours after birth. They observe that longitudinal decline of intact HIV-1 proviruses during ART is associated with antiviral NK cell immune responses.

Hartana et al., 2022, Cell Reports *40*, 111126
July 19, 2022 © 2022 The Author(s).
https://doi.org/10.1016/j.celrep.2022.111126

CellPress

Cell Reports

CellPress
OPEN ACCESS

Report

Immune correlates of HIV-1 reservoir cell decline in early-treated infants

Ciputra Adijaya Hartana,[1,2] Pilar Garcia-Broncano,[1,2] Yelizaveta Rassadkina,[1] Xiaodong Lian,[1,2] Chenyang Jiang,[1,2] Kevin B. Einkauf,[1] Kenneth Maswabi,[4] Gbolahan Ajibola,[4] Sikhulile Moyo,[4] Terence Mohammed,[4] Comfort Maphorisa,[4] Joseph Makhema,[4] Yuko Yuki,[5,6] Maureen Martin,[5,6] Kara Bennett,[7] Patrick Jean-Philippe,[8] Mathias Viard,[5,6] Michael D. Hughes,[9] Kathleen M. Powis,[3,4,9,10] Mary Carrington,[1,5,6] Shahin Lockman,[2,3,4] Ce Gao,[1] Xu G. Yu,[1,2,3] Daniel R. Kuritzkes,[2,3] Roger Shapiro,[3,4,9] and Mathias Lichterfeld[1,2,3,11,*]

[1]Ragon Institute of MGH, MIT and Harvard, Cambridge, MA 02139, USA
[2]Division of Infectious Diseases, Brigham and Women's Hospital, Boston, MA 02115, USA
[3]Harvard Medical School, Boston, MA 02115, USA
[4]Botswana – Harvard AIDS Institute Partnership, Gaborone, Botswana
[5]Basic Science Program, Frederick National Laboratory for Cancer Research, National Cancer Institute, Frederick, MD 20892, USA
[6]Laboratory of Integrative Cancer Immunology, Center for Cancer Research, National Cancer Institute, Bethesda, MD 20892, USA
[7]Bennett Statistical Consulting, Inc., Ballston Lake, NY 12019, USA
[8]Division of AIDS, NIAID, NIH, Rockville, MD 20852, USA
[9]Department of Immunology and Infectious Diseases, Harvard T.H. Chan School of Public Health, Boston, MA 02115, USA
[10]Department of Medicine and Pediatrics, Massachusetts General Hospital, Boston, MA 02114, USA
[11]Lead contact
*Correspondence: mlichterfeld@partners.org
https://doi.org/10.1016/j.celrep.2022.111126

SUMMARY

Initiation of antiretroviral therapy (ART) in infected neonates within hours after birth limits viral reservoir seeding but does not prevent long-term HIV-1 persistence. Here, we report parallel assessments of HIV-1 reservoir cells and innate antiviral immune responses in a unique cohort of 37 infected neonates from Botswana who started ART extremely early, frequently within hours after birth. Decline of genome-intact HIV-1 proviruses occurs rapidly after initiation of ART and is associated with an increase in natural killer (NK) cell populations expressing the cytotoxicity marker CD57 and with a decrease in NK cell subsets expressing the inhibitory marker NKG2A. Immune perturbations in innate lymphoid cells, myeloid dendritic cells, and monocytes detected at birth normalize after rapid institution of antiretroviral therapy but do not notably influence HIV-1 reservoir cell dynamics. These results suggest that HIV-1 reservoir cell seeding and evolution in early-treated neonates is markedly influenced by antiviral NK cell immune responses.

INTRODUCTION

Despite remarkable advances in prevention of vertical HIV-1 transmission (Van de Perre et al., 2021) and antiretroviral drug development, pediatric HIV-1 infection remains a difficult-to-treat disease that occurs in approximately 2 million children worldwide (UNAIDS, 2019). Antiretroviral treatment (ART) of neonates, infants, and children still depends, to a large extent, on legacy drugs that require more frequent dosing and are associated with a less favorable side-effect profile, whereas more convenient and better tolerated combination regimens have not been formulated for pediatric use. Moreover, ART adherence challenges are frequent among pediatric patients and often increase the risk for treatment failures. For these reasons, pediatric HIV-1 infection is commonly associated with suboptimal clinical outcomes (Iyun et al., 2020; Kuhn et al., 2020; Mutanga et al., 2019; Technau et al., 2018); modifications and improvements of treatment interventions for this specific patient group represent an important research priority.

Despite these difficulties, pediatric HIV-1 infection may also represent a notable opportunity to explore immune mechanisms, host factors, and treatment modalities that may support or facilitate a drug-free remission or functional cure of HIV-1 infection. Indeed, a number of previous reports suggest that spontaneous control of HIV-1 in pediatric patients is possible (Frange et al., 2016; McMahon et al., 2017; Violari et al., 2019), although viral rebound has occurred in some cases after many months or years of drug-free control (Luzuriaga et al., 2015). The mechanisms influencing the establishment, evolution, and long-term persistence of HIV-1 reservoir cells in neonates may differ profoundly from adults, due to the specific characteristics of immune cells in the developing immune system of newborn infants. For example, T cells and natural killer (NK) cells from neonates tend to display weaker cytotoxic activities but seem to have lower thresholds for activation by innate cytokines (Ivarsson et al., 2013; Simon et al., 2015). In addition, CD4 T cells in neonates are frequently polarized toward a T helper type 2 (Th2) and a regulatory T cell profile (Rudd, 2020), a propensity that

This is an open access article under the CC BY license (http://creativecommons.org/licenses/by/4.0/).

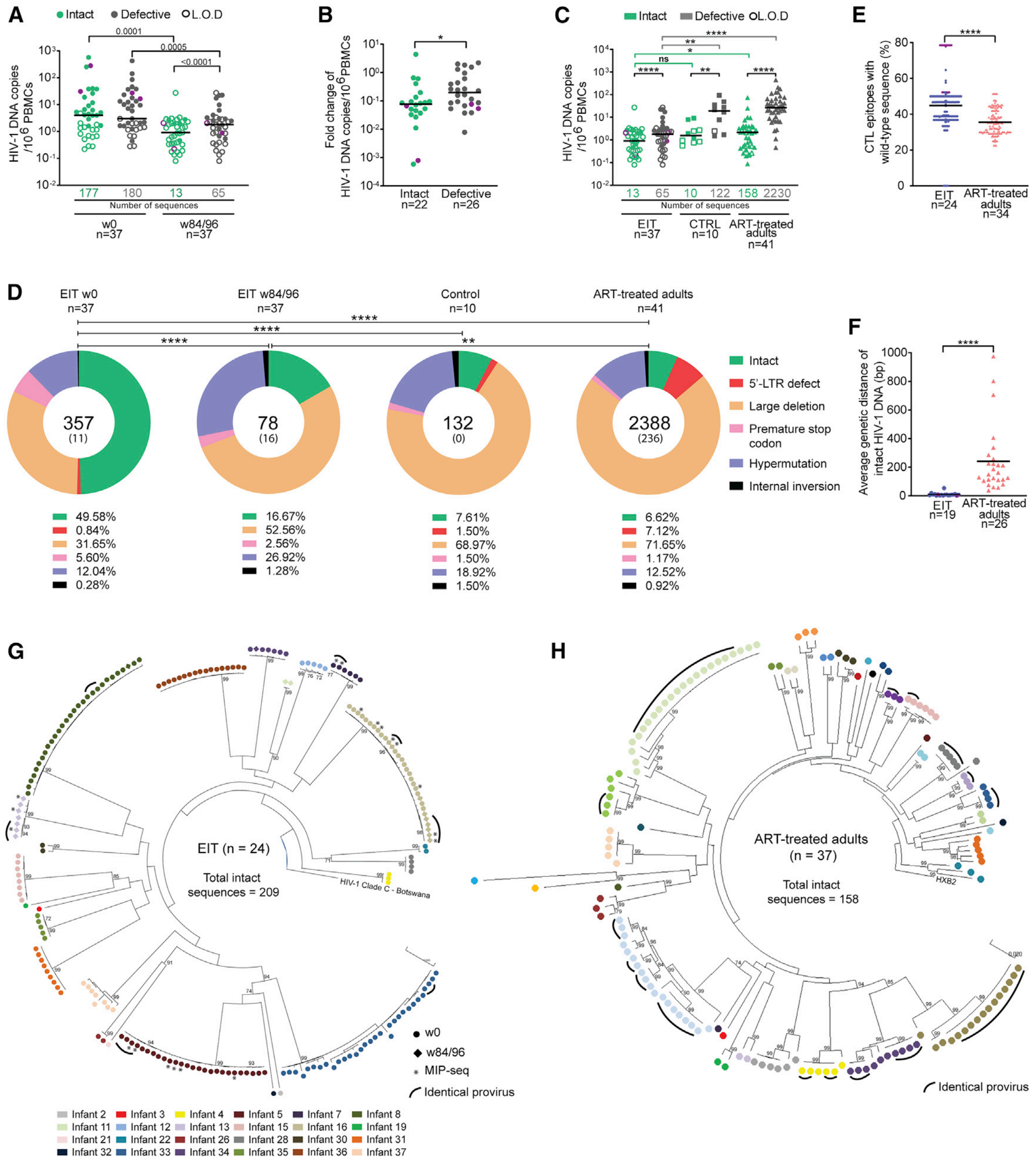

Figure 1. Distinct viral reservoir landscape in early-treated HIV-1-infected infants

(A) Frequency of intact and defective proviruses in early-treated HIV-1-infected infants at week 0 after birth (n = 37) and week 84/96 (n = 37). Limit of defection (LOD) was calculated as 0.5 copies per maximum number of cells tested without target identification. The total number of intact and defective sequences is shown under the x-axis.

(B) Fold change in proportion of intact (n = 22) and defective (n = 26) proviruses between baseline (week 0) and week 84/96 in early-treated infants. Data from all infants with detectable proviruses at baseline were included.

(C) Frequency of intact and defective proviruses in early-treated infants (EIT) at week 84/96 (n = 37), in control infants who started ART at a median of 125 days (range: 79–350 days) after birth (CTRL) (n = 10), and in HIV-1-infected adults who have been treated with ART for an average of 13 years (n = 41). The total number of intact and defective sequences is shown.

(D) Pie charts reflecting the contribution of intact and defective proviruses to the total number of proviruses detected in each cohort; time points of analysis are as in (C). Total number of proviruses is shown in the center of each pie chart, with the number of identical proviruses shown in brackets. In ART-treated adults, only near-full-length amplification products were sequenced.

(E) Proportion of CTL epitopes (restricted by autologous HLA class I alleles) within intact proviruses that display the clade C wild-type sequence (for clade C-infected infants from Botswana) (n = 24) or the clade B wild-type sequences (for clade B-infected adults from the US) (n = 34).

(legend continued on next page)

may be of benefit for resisting inflammatory complications associated with common childhood viral illnesses but which may support viral reservoir persistence; indeed, in non-human primate models of retroviral infections, higher levels of simian immunodeficiency virus (SIV) DNA were observed in CTLA-4^{+} PD-1^{-} memory CD4 T cells, which share phenotypic markers with regulatory T cells (McGary et al., 2017).

Current guidelines recommend initiation of ART in all HIV-1-infected infants at the time of diagnosis, independent of clinical, virological, or immunological characteristics (WHO, 2010), but in reality, ART commencement in children in sub-Saharan countries is frequently delayed because diagnostic testing generally occurs 6 weeks after birth (Shiau et al., 2018). Nevertheless, there is a growing consensus that immediate or very early ART initiation can translate into important health benefits for neonates with HIV-1, although the precise effects and consequences of early ART in infected neonates have only been systematically studied in small numbers of patients (Garcia-Broncano et al., 2019; Kuhn et al., 2021; Maswabi et al., 2021). The Early Infant Treatment (EIT) study, a prospective clinical trial in Botswana, was designed to evaluate clinical, immunological, and virological effects of early ART initiation in newborns infected with HIV-1. The participants of this study were tested for HIV-1 infection at the time of birth, started on ART within an average of 1–2 days after HIV-1 diagnosis, and longitudinally followed during the subsequent 2 years, with periodical sampling of peripheral blood mononuclear cells (PBMCs) for immunological and virological testing. Here, we analyzed host immune mechanisms associated with viral reservoir decline in this specific patient population.

RESULTS

HIV-1 reservoir evolution in early-treated infants

Between 2015 and 2018, a total of n = 40 infants were enrolled in the antepartum cohort of the EIT study, with a positive HIV-1 DNA PCR reaction from samples collected at a median of 1 day from birth (range: 0–4 days). Two additional children were diagnosed with peripartum infection, characterized by an HIV-1 DNA PCR that was negative at birth but positive at day 30 and 42, respectively. Two study participants died, and three others were not yet analyzed at the time of this report, leading to data from n = 37 (35 with intrauterine and 2 with peripartum infection) being available for analysis; data from n = 10 (9 with intrauterine infection and one with peripartum infection) of these study participants were already reported in an earlier manuscript (Garcia-Broncano et al., 2019) (Table S1). Infants with intrauterine HIV-1 infection received antiretroviral prophylaxis consisting of nevirapine and zidovudine (±lamivudine) within a median of 7 h after birth, and then converted to treatment doses of nevirapine, zidovudine, and lamivudine upon enrollment into the EIT study at a median of 2 days (range: 1–5 days); after at least 2 weeks (and 40 weeks gestational age equivalence), infants were switched to a combination of ritonavir-boosted lopinavir, lamivudine, and zidovudine, consistent with the study protocol (Garcia-Broncano et al., 2019). The two infants with peripartum infection started treatment 31 and 50 days after birth, respectively. Most infants initially responded to ART and developed durable viral load suppression (Figure S1A); however, transient viral rebound after initial suppression was noted in many infants, likely reflecting medication non-adherence and highlighting the difficulties of effectively treating neonatal HIV-1 infection with currently available pharmacological agents. A total of n = 10 HIV-1-infected infants from Botswana who started ART at later time points (median of 125 days [range: 79–350 days] after birth [Table S1]) were studied as controls; these infants were described in our previous work (Garcia-Broncano et al., 2019).

To analyze the dynamics of HIV-1 reservoir cell evolution in this study cohort, we used near-full-length individual proviral next-generation sequencing (FLIP-seq), an approach that allows us to distinguish intact from defective proviruses, infer clonality based on proviral sequence identity, and evaluate sequence variations consistent with mutational escape from antiviral immune responses (Lee et al., 2017). Using approximately 2–3 million PBMCs available from each study participant, we were able to detect intact HIV-1 sequences at baseline in n = 22 study participants and defective proviruses in 26 study participants (Figures 1A and 1B). After 84–96 continuous weeks of treatment, proviral DNA levels had decreased by 5- to 10-fold; this decrease was significantly more pronounced for intact HIV-1 proviruses, which accounted for approximately half of all detected proviruses at baseline but were profoundly underrepresented after the 84/96-week treatment period, when they contributed less than 20% of all proviral species (Figures 1A, 1B, and 1D). Notably, intact and defective proviruses from early-treated infants after approximately 2 years of treatment were significantly lower than in a cohort of adult ART-treated HIV-1 patients (recruited in the US) who had remained on suppressive ART for an average of 13 years (Figure 1C); they also tended to be lower compared with the control cohort of HIV-1-infected infants with later treatment initiation. However, this trend did not reach statistical significance for intact proviruses (Figure 1C), possibly due to uncertainties in intact proviral reservoir quantification at the limit of detection in the small numbers of PBMCs available from infants. Notably, in multiple study participants, we detected intact proviruses that were completely sequence identical; such

(F) Average genetic distance of intact proviruses from early-treated infants (n = 19) and ART-treated adults (n = 26), determined by pairwise comparisons between all intact sequences within each study person. Data from all EIT with at least two different intact proviruses were included.

(G and H) Circular maximum-likelihood phylogenetic trees of intact proviral sequences from early-treated infants and ART-treated adults. HXB2 was used as reference sequence for clade B; a clade C HIV-1 sequence from Botswana was used as reference for clade C. Clonal sequences, defined by complete sequence identity are highlighted by black arches. Bootstrap analysis with 1,000 replicates was performed to assign confidence to tree nodes; bootstrap support values >70% are shown in the trees. *p < 0.05, **p < 0.01, ****p < 0.0001.

Two-tailed Mann-Whitney U tests were used for data shown in (B), (E), and (F); Wilcoxon matched-pairs signed-rank test were used for data shown in (A) and (C); Kruskal Wallis test with post-hoc Dunn's test were used for data shown in (C); chi-square test was used for data shown in (D). (A–C, E, and F) Data from infants with peripartal infection are indicated in purple.

clusters were observed at baseline (prior to initiation of antiviral therapy) and at week 84 and result from clonal proliferation of infected cells that pass on their identical proviral sequences during cell divisions (Figure 1G). Clusters of clonally expanded intact proviruses were also observed in our adult comparison cohort (Figure 1H), consistent with prior findings (Hiener et al., 2017; Lee et al., 2017).

Intact proviruses from early-treated infants displayed significantly reduced phylogenetic diversity compared with the adult reference cohort (Figures 1F–1H) and showed limited evidence for sequence adaptation to cytotoxic T lymphocyte (CTL)- or antibody-driven immune selection pressure (Figures 1E, S1B, and S1C). Almost all intact proviral sequences detected in EIT study participants showed tropism for CCR5 co-receptor usage, a marked contrast to ART-treated adults (Figure S1D). Notably, the proportion of hypermutated sequences in EIT study participants was not significantly different from ART-treated adults at any of the analyzed time points (Figure 1D); however, the number of sequences displaying simultaneous evidence of both A3G and A3F-induced hypermutations was larger in adults compared with infants (Figure S1E). Together, these results demonstrate that very early initiation of ART in neonates induces a distinct viral reservoir profile, characterized by small numbers of intact proviruses with limited evidence of sequence evolution or adaptation to host immune responses.

HIV-1 reservoir decline correlates with phenotypic changes in NK cells

The reduction of intact proviral sequences in early-treated neonates may reflect immune-mediated effects that lead to specific killing of reservoir cells with higher vulnerability to host immune mechanisms. Since HIV-1-specific T and B cell responses are typically low in breadth and magnitude in infected neonates (Ananworanich et al., 2014; Garcia-Broncano et al., 2019; Rinaldi et al., 2020), specifically when antiretroviral therapy is instituted during very early stages of infection, we focused on NK cells, the main effector cell component of the innate immune system, to explore immune mechanisms influencing the trajectory of viral reservoir cells in infants. Overall, the phenotypic profile of NK cells showed profound global changes during the postnatal period (Figures 2A and S3A–S3C). In particular, we observed that $CD57^+$ NK cells, characterized by elevated antiviral and cytotoxic activities (Lopez-Verges et al., 2010), tended to increase over time after birth, consistent with the physiologic maturation of the innate immune system; this trend was most obvious in $CD16^-$ $CD56^{dim}$ NK cells (mostly known for antiviral effects through cytokine secretion) and in $CD16^{dim}$ $CD56^{dim}$ NK cells (characterized by enhanced cytotoxic properties) (Figures 2B–2D). Notably, longitudinal increases in the proportions of $CD16^-$ $CD56^{dim}$ and $CD16^{dim}$ $CD56^{dim}$ cells within the $CD57^+$ NK cell pool were correlated with a reciprocal decline in the frequencies of intact proviruses between baseline and week 84, suggesting an active role of these cells in restricting persistence of HIV-1 reservoir cells (Figure 2F). In contrast, proportions of $CD16^-$ $CD56^{dim}$ and $CD16^{dim}$ $CD56^{dim}$ cells expressing NKG2A, an inhibitory NK cell marker (Houchins et al., 1997; Kamiya et al., 2019), decreased over time (Figures 2B, 2C, and 2E). The longitudinal reduction of intact HIV-1 proviruses was most pronounced in patients with the strongest decline of $NKG2A^+$ NK cells and least obvious in persons with limited or no longitudinal decrease of $NKG2A^+$ NK cells (Figure 2G), implying that inhibitory signals in innate immune cells may increase host susceptibility to viral reservoir cell persistence or expansion. We also noted a trend for a stronger decline of intact proviruses in carriers of HLA-A alleles known to be expressed at lower levels, consistent with previous observations of better control of HIV-1 viral load and slower disease progression among subjects with lower HLA-A expression levels in natural history cohorts of HIV-1-infected adults (Ramsuran et al., 2018) (Figure S3D). This trend was most apparent in the subgroup of patients carrying the HLA-B −21MT genotype (Figure S3E), which is associated with enhanced expression of HLA-E, the ligand for NKG2A, and with decreased cytotoxic activities of $NKG2A^+$ NK cells toward HIV-1-infected target cells (Merino et al., 2013; Ramsuran et al., 2018).

For a more detailed analysis of the $CD16^{dim}$ $CD56^{dim}$ NK cells and their association with viral reservoir evolution, we conducted a computational exploration of their phenotypic profile (Figures 3A–3C). Using FlowSOM for identifying concatenated subsets of cells within this specific NK cell population, we distinguished a total of seven phenotypically distinct clusters. This approach identified a distinct subset of NK cells, characterized by elevated expression of the inhibitory NK cell markers NKG2A, KLRD1 (CD94) (Brooks et al., 1997), and Siglec-7 (Nicoll et al., 1999) (cluster 2) that decreased after birth; in contrast, an NK cell subpopulation defined by upregulation of NK cell activation markers CD57 and the activating NK cell receptors NKG2C (cluster 3) dynamically expanded over time (Figure 3D). Notably, the increase of activated (cluster 3) and the decrease of inhibitory (cluster 2) NK cell populations were statistically associated with the longitudinal reduction of intact proviruses (Figure 3E), further supporting the hypothesis that intact proviral reservoir decline is influenced by antiviral activities of NK cells. Associations between other NK cell subpopulations and intact viral reservoir cell dynamics were less obvious (Figures S4A and S4B). Although our work is limited to phenotypic evaluations and (due to the lack of sufficient PBMC samples available from neonates) does not involve functional immunologic assays, our results suggest that intact proviral reservoir evolution in ART-treated infants is a dynamic process critically influenced and modulated by specific subsets of NK cells.

Innate lymphoid cells and dendritic cells in early-treated neonates

To explore whether additional innate immune cells correlated with viral reservoir evolution in children, we investigated proportions of innate lymphoid cells (ILCs) (Figure S5A) in our study patients. We observed that all three known classes of ILCs (ILC1, ILC2, ILC3) (Eberl et al., 2015) were significantly reduced in HIV-1-infected infants at birth, relative to a control cohort of HIV-1-uninfected infants (Figure 4A); however, these differences were no longer visible at subsequent time points of follow up, suggesting that rapid initiation of antiretroviral therapy in HIV-1-infected neonates can normalize ILC homeostasis (Figure 4A). A similar observation was made for type 2/3 myeloid dendritic cells (mDCs) (which are endowed with enhanced abilities to

CellPress
OPEN ACCESS

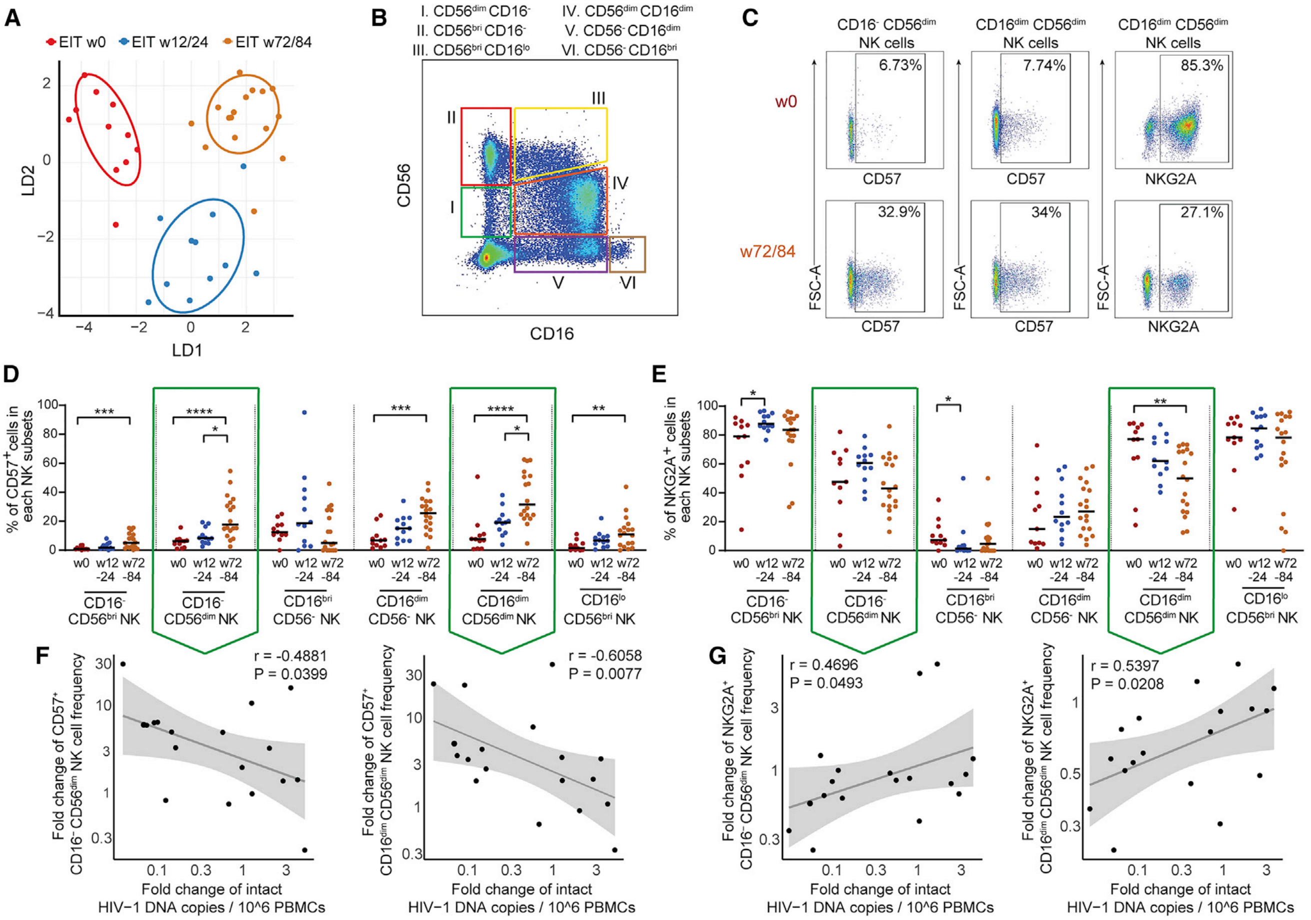

Figure 2. Longitudinal evolution of NK cell responses correlates with trajectory of intact HIV-1 proviruses

(A) Linear discriminant analysis of the phenotypic profile of NK cells responses at indicated time points in early-treated infants. NK cells were phenotypically characterized using flow cytometry with 9 distinct surface markers.

(B) Representative flow cytometry pseudocolor plot highlighting subclassification of NK cell subsets stratified according to CD16 and CD56 expression.

(C) Pseudocolor plots indicating expression of CD57 and NKG2A in indicated NK cell subsets at week 0 (immediate after birth) and at 72/84 weeks after birth.

(D and E) Longitudinal evolution of CD57-expressing (D) and NKG2A-expressing (E) NK cell subsets in early-treated infants. Data from weeks 0 (n = 11), 12/24 (n = 12), and 72/84 (n = 18) are shown. $^{*}p < 0.05$, $^{**}p < 0.01$, $^{***}p < 0.001$, $^{****}p < 0.0001$; Kruskal Wallis test with post-hoc Dunn's test.

(F and G) Correlation between proportional changes of indicated NK cell subsets (between weeks 0 and 72/84) and corresponding changes in intact HIV-1 proviruses. Spearman correlation coefficient is indicated.

stimulate T cells and can phenotypically be characterized by surface expression of CD1c [Villani et al., 2017]) and plasmacytoid DCs (pDCs); relative to HIV-1-uninfected patients, both of these cell types were significantly reduced in HIV-1-infected infants at birth but not at subsequent follow-up time points (Figure 4B). Additionally, more limited changes between early-treated infants and HIV-1-negative neonates were observed for other DC subtypes (Figures S5B and S5C). Notably, the proportion of classical monocytes ($CD14^{dim}$ $CD16^{-}$) was reduced at birth in HIV-1-infected infants, while intermediate ($CD14^{dim}$ $CD16^{dim}$) and non-classical ($CD14^{low/-}$ $CD16^{bright}$) monocytes (Figure S5A) were expanded; these disruptions in HIV-1-infected infants also appeared to normalize at subsequent analysis time points following institution of antiretroviral therapy (Figure 4C). No statistical associations were noted between frequencies of ILCs, DCs, or monocytes and the longitudinal decline of intact proviruses, suggesting that these innate immune cells do not directly influence proviral reservoir changes in infected neonates.

DISCUSSION

Understanding the long-term persistence and evolution of HIV-1 reservoir cells is arguably most impactful in vertically infected children who, in the absence of curative interventions, will require ART for their entire lifespan. Using longitudinal PBMCs collected from children who started ART within a few hours/days after birth, we performed a detailed investigation of viral reservoir establishment in neonates and interrogated host factors in the developing immune system that may influence the subsequent evolution of viral reservoir cell pool size. We found that early ART initiation resulted in remarkably few numbers of intact proviruses, with very little evidence of sequence

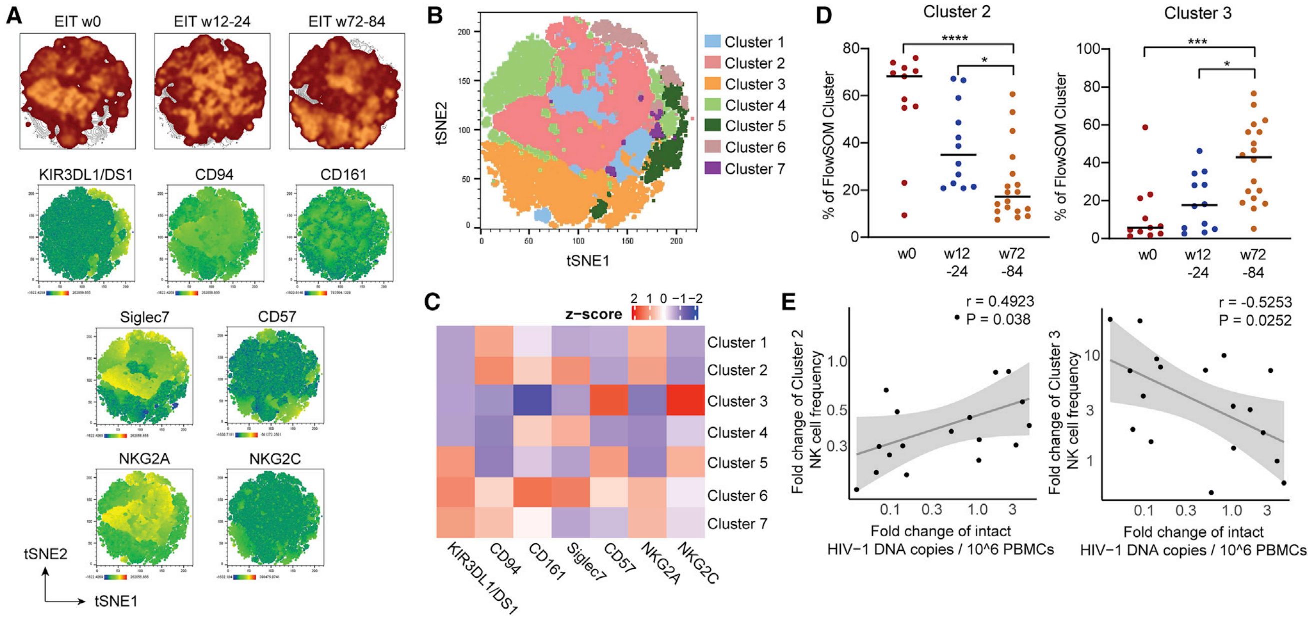

Figure 3. NK cell subsets associated with longitudinal trajectory of intact proviruses

(A) (Top) Global t-distributed stochastic neighbor embedding (tSNE) maps of CD56^{dim} CD16^{dim} NK cells from early-treated infants; data from indicated time points are shown separately as overlays. (Bottom) tSNE maps showing the expression of individual phenotypic markers measured by flow cytometry in concatenated CD56^{dim} CD16^{dim} NK cells analyzed from early-treated infants.

(B) tSNE map displaying 7 phenotypically distinct clusters identified by FlowSOM within concatenated CD56^{dim} CD16^{dim} NK cells from early-treated infants.

(C) Heatmap showing the mean fluorescence intensity (MFI) of the phenotypic parameters measured in the 7 clusters shown in (B).

(D) Longitudinal evolution of clusters 2 and 3 NK cell populations from weeks 0 (n = 11), 12/24 (n = 12), and 72/84 (n = 18). *p < 0.05, ***p < 0.001, ****p < 0.0001; Kruskal Wallis test with post-hoc Dunn's test.

(E) Correlation between longitudinal fold changes in proportions of cluster 2 or 3 NK cells (between weeks 0 and 72/84) and corresponding changes in intact proviruses from early-treated infants. Spearman correlation coefficient is shown.

diversification or immune adaptation to host major histocompatibility complex (MHC) class I alleles. Following ART initiation, viral reservoir cell evolution in neonates with HIV-1 seemed to be modulated by innate host factors, although due to limited numbers of cells available for investigation, no functional studies could be conducted to support this observation. In particular, NK cell subpopulations expressing activating phenotypic markers appeared to restrict HIV-1 reservoir cell pool size during ART, presumably through their ability to sense virally infected cells and eliminate them by direct cytotoxic effects. In contrast, NK cells expressing the inhibitory NK cell marker NKG2A were inversely associated with the longitudinal decline of intact HIV-1 proviruses. Collectively, our results suggest that in neonates, HIV-1 reservoir cell dynamics may be influenced by innate NK cell responses; future studies will be necessary to determine whether such early innate immune responses will leave a durable footprint in the proviral reservoir profile at more advanced stages of infection.

The longitudinal analysis of proviral species, reported here in a relatively large cohort of children with HIV-1 followed from birth, can help to dissect specific characteristics of viral reservoir cell dynamics in early infancy. While ART can effectively restrict refueling of the viral reservoir cell pool through new infection of HIV-1-negative cells, it has no activity against already infected cells; the kinetic decline of infected cells after institution of ART likely reflects dying of infected cells, either through natural decay, through cell-intrinsic viral cytopathic effects, or through active killing by immune effector cells. These mechanisms may be antagonized by proliferation of infected cells, a process that can expand the viral reservoir cell pool size and may be driven by homeostatic proliferative cytokine signals (Chomont et al., 2009), by cell-autonomous activation of cell proliferation through proviral insertional mutagenesis in oncogenes (Liu et al., 2020), or by antigen-specific stimulation of T cell proliferation (Mendoza et al., 2020; Simonetti et al., 2021). This last mechanism may be of particular relevance during the immediate postnatal period, when the antigen-inexperienced neonatal immune system is exposed to massive amounts of foreign antigens after new-onset bacterial and viral colonization of the gastrointestinal and respiratory tracts (Palmer et al., 2007). In our study, several clusters of sequence-identical intact proviral species were already detected at birth, indicating that clonal proliferation of infected cells can likely occur during fetal development, possibly reflecting antigen-specific T cell proliferation driven by recently discovered microbial communities in the prenatal intrauterine environment (Aagaard et al., 2014; Collins, 2014); moreover, fetal T cells typically are constitutively in high proliferative states and tend to be exquisitely responsive to the homeostatic cytokine interleukin-7 (IL-7) (Schonland et al., 2003).

While a faster decline of intact HIV-1 proviruses relative to defective proviral species has been reported in a number of recent studies in adults (Falcinelli et al., 2020; Gandhi et al.,

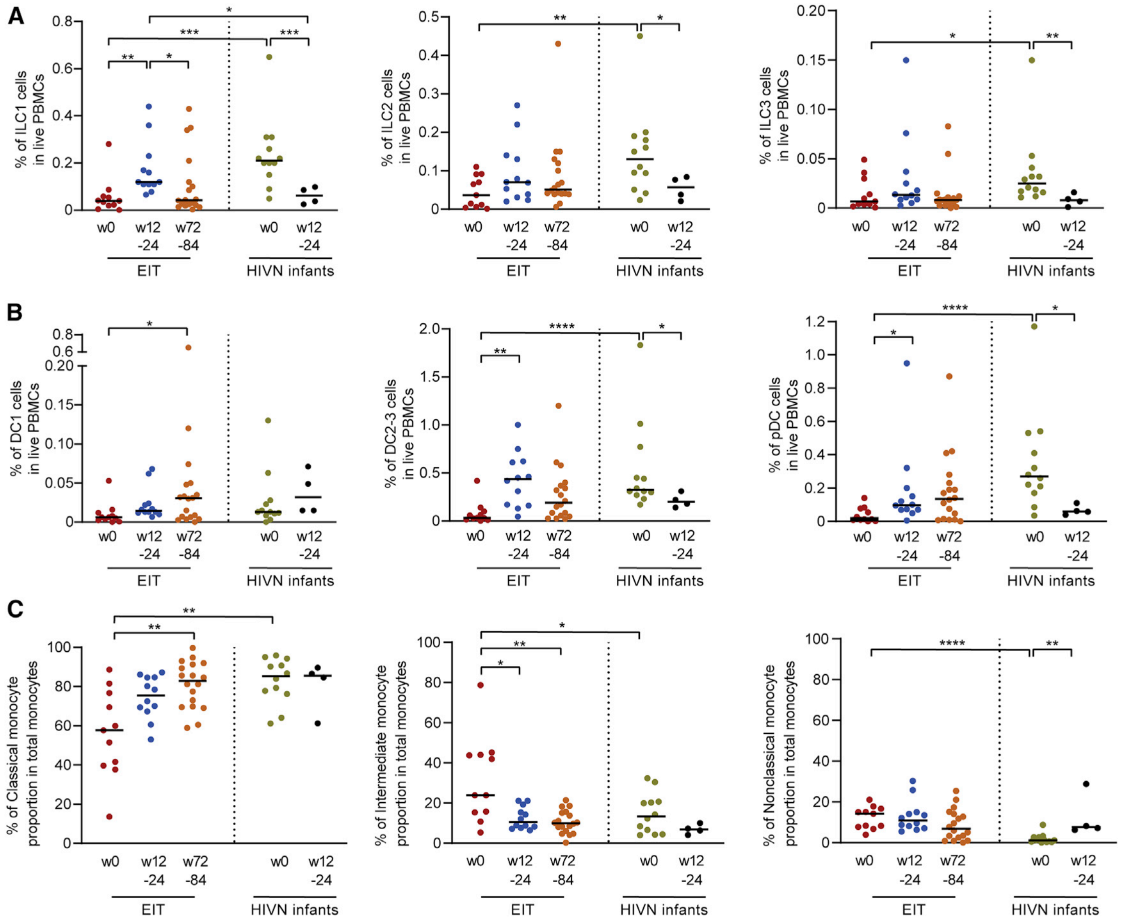

Figure 4. Innate immune cell profile in early-treated infants

Proportion of ILC1, ILC2, and ILC3 (A) or DC1, DC2/3, and plasmacytoid dendritic cells (pDCs) (B) and of monocyte populations (C) within all PBMCs at weeks 0 (n = 11), 12/24 (n = 12), and 72/84 (n = 18) in early-treated HIV-1-infected infants (EIT) and at weeks 0 (n = 12) and 12/24 (n = 4) in HIV-1-negative infants. *$p < 0.05$, **$p < 0.01$, ***$p < 0.001$; Kruskal Wallis test with post-hoc Dunn's test and Mann Whitney U test adjusted for multiple testing.

2020; Peluso et al., 2020), immune correlates of viral reservoir decline remain largely undefined. In this study, we provide a detailed parallel analysis of immune responses that are associated with the reduction of intact proviruses after birth. Through synchronized assessments of virological and immunological parameters during defined longitudinal follow-up time points, we demonstrate a statistical association between declining numbers of intact HIV-1 proviruses and reciprocal increases in $CD57^+$ NK cell subpopulations endowed with increased cytotoxic activities, while NK cell subsets expressing inhibitory NK cell markers showed an opposite pattern. Notably, no such associations were noted between NK cell populations and defective proviruses, which generally showed a slower and weaker longitudinal decline. Together, our data suggest an important role of innate effector cells for viral reservoir decline in neonates. Notably, a more important role of NK cells, relative to HIV-1-specific T cells, for controlling HIV-1 replication, has been suggested in recent studies involving ART-naïve African children (Vieira et al., 2021). Moreover, our observations seem to resonate well with recent findings demonstrating a longitudinal decline of intact HIV-1 proviruses during administration of a TLR7 agonist to ART-treated HIV-1 controllers (SenGupta et al., 2021), which was associated with transcriptional signatures suggesting enhanced DC and NK cell cross-talk and an increase in the cytotoxic potential of NK cells after TLR7 agonist dosing. Notably, functionally enhanced innate immune cell responses in persons with natural HIV-1 immune control have been reported in a number of recent studies (Hartana et al., 2021; Marras et al., 2013; Martin-Gayo et al., 2018), further supporting a possible role of innate immunity in restricting HIV-1 replication or controlling viral reservoir cells. Clearly, a closer mechanistic evaluation of how innate immune responses may sense, monitor, or target HIV-1-infected cells in ART-treated patients is a high research priority; moreover, analyzing specific functional, transcriptional, or epigenetic features of neonatal NK cells that are associated with the decline of intact HIV-1 proviruses will be of interest.

Limitations of the study

PBMC samples that can be collected from neonates are extremely limited, frequently precluding additional immunophenotypic and functional immune assessments in these study persons; specifically, HIV-1-specific T cells could not be assessed

in the majority of patients described here, although data on HIV-1-specific T cells were reported in a subset of study patients before (Garcia-Broncano et al., 2019). Moreover, limitations of available PBMC samples did not allow us to determine how alternative microbial pathogens may influence HIV-1 reservoir cell evolution and innate host immune mechanisms in infants; this is particularly true for cytomegalovirus (CMV), with which most African infants are infected during early infancy, independently of HIV-1 infection or exposure (Gompels et al., 2012; Hsiao et al., 2013).

STAR★METHODS

Detailed methods are provided in the online version of this paper and include the following:

- KEY RESOURCES TABLE
- RESOURCE AVAILABILITY
  - Lead contact
  - Materials availability
  - Data and code availability
- EXPERIMENTAL MODEL AND SUBJECT DETAILS
- METHOD DETAILS
  - Sample processing
  - HIV-1 near-full-genome sequencing
  - Flow cytometry
- QUANTIFICATION AND STATISTICAL ANALYSIS

SUPPLEMENTAL INFORMATION

Supplemental information can be found online at https://doi.org/10.1016/j.celrep.2022.111126.

ACKNOWLEDGMENTS

The authors gratefully acknowledge the support of all study individuals and their families. The authors also acknowledge the Botswana Ministry of Health; the members of the Institutional Review Boards in Botswana and at the Harvard School of Public Health; the members of the NIH Data Monitoring and Safety Board (Haroon Saloojee [Chair], Jerrold J. Ellner, David P. Harrington, Grace John-Stewart, Catherine Hill, Steven Joffe, Alwyn Mwinga, Andrew J. Nunn, Merlin L. Robb); the MGH DNA core facility; the National Institutes of Health (Judi Miller, Ellen Decarlo, Sheryl Zwerski, P.J.-P.); and staff members at the Harvard TH Chan School of Public Health and the Botswana-Harvard Partnership, in particular Tlhabologo Baitseme, Thelma Ketshabile, Maduo Oabona, Mogomotsi Modikwa, Salome Othusitse, Obakeng Makalane, Rabana Thuto Aeshar, Maipelo Kegakilwe, Nametsegang Tshosa, Dorcus Babuile, Jamie Ramaabya, Simon Mosopa, Muchaneta Bhondai, Kebaiphe Moabi, Tebogo Mokotedi, Boitumelo Tebape, Kesegofetse Agisanang, Masego Rabantetse, Disaro Jack, Tumalano Sekoto, Thabani Ncube, Lorato Esele, Princess Bobo Mapenshi, Tshepho Frank, Ria Madison, Mompati Mmalane, Max Essex, Mogomotsi Matshaba, Loeto Mazhani, Madisa Mine, Chipo Petlo, Lucia Ricci, and Lendsey Melton. This project was supported by NIH grant U01 AI114235 (to R.S., D.R.K., M.L.) and NIH grant AI120850 (to K.M.P.). M.L. is supported by NIH grants AI098487, AI135940, AI114235, AI117841, AI120008, AI152979, AI155233, and DK120387. X.G.Y. is supported by NIH grants AI116228, AI078799, HL134539, AI125109, and DA047034. M.L. and X.G.Y. are supported by the Bill and Melinda Gates Foundation (INV-002703). M.L. and R.S. are members of the NIH-funded P.A.V.E. Martin Delaney Collaboratory (UM1AI164566). M.L. is a member of the EPIICAL Consortium, funded by ViiV Healthcare. This project has been funded in whole or in part with federal funds from the Frederick National Laboratory for Cancer Research under contract no. HHSN261200800001E. The content of this publication does not necessarily reflect the views or policies of the Department of Health and Human Services, nor does mention of trade names, commercial products, or organizations imply endorsement by the US government. This research was supported in part by the Intramural Research Program of the NIH, Frederick National Lab, Center for Cancer Research.

AUTHOR CONTRIBUTIONS

Concept, design, and discussion, C.A.H., P.G.B., X.G.Y., P.J.-P., R.S., D.R.K., and M.L.; whole-genome amplification and HIV-1 sequencing, P.G.B., Y.R., and K.B.E.; integration site analysis, X.L. and C.J.; immune phenotyping and immunology assays, C.A.H., P.G.B., and Y.R.; bioinformatics analysis, C.G.; conduction of EIT clinical trial, K.M., G.A., S.M., T.M., C.M., J.M., K.B., K.M.P., S.L., and R.S.; data interpretation, analysis, and presentation, C.A.H., X.L., C.J., C.G., M.V., M.C., R.S., X.G.Y., and M.L.; supervision of immunological and virological experiments, M.L.; manuscript writing, review, and editing, C.A.H., R.S., D.R.K., X.G.Y., M.C., S.L., K.M.P., and M.L.

DECLARATION OF INTERESTS

D.R.K. has received consulting honoraria and/or research support from Gilead, Merck, and ViiV. M.L. has received speaking and consulting honoraria from Merck.

INCLUSION AND DIVERSITY

We worked to ensure gender balance in the recruitment of human subjects. One or more of the authors of this paper self-identifies as an underrepresented ethnic minority in science. One or more of the authors of this paper self-identifies as a member of the LGBTQ+ community. One or more of the authors of this paper received support from a program designed to increase minority representation in science. While citing references scientifically relevant for this work, we also actively worked to promote gender balance in our reference list.

Received: December 13, 2021
Revised: April 13, 2022
Accepted: June 30, 2022
Published: July 19, 2022

REFERENCES

Aagaard, K., Ma, J., Antony, K.M., Ganu, R., Petrosino, J., and Versalovic, J. (2014). The placenta harbors a unique microbiome. Sci. Transl. Med. *6*, 237ra265.

Ananworanich, J., Puthanakit, T., Suntarattiwong, P., Chokephaibulkit, K., Kerr, S.J., Fromentin, R., Bakeman, W., Intasan, J., Mahanontharit, A., Sirivichayakul, S., et al. (2014). Reduced markers of HIV persistence and restricted HIV-specific immune responses after early antiretroviral therapy in children. AIDS *28*, 1015–1020.

Bricault, C.A., Yusim, K., Seaman, M.S., Yoon, H., Theiler, J., Giorgi, E.E., Wagh, K., Theiler, M., Hraber, P., Macke, J.P., et al. (2019). HIV-1 neutralizing antibody signatures and application to epitope-targeted vaccine design. Cell Host Microbe *26*, 296.

Brooks, A.G., Posch, P.E., Scorzelli, C.J., Borrego, F., and Coligan, J.E. (1997). NKG2A complexed with CD94 defines a novel inhibitory natural killer cell receptor. J. Exp. Med. *185*, 795–800.

Chomont, N., El-Far, M., Ancuta, P., Trautmann, L., Procopio, F.A., Yassine-Diab, B., Boucher, G., Boulassel, M.R., Ghattas, G., Brenchley, J.M., et al. (2009). HIV reservoir size and persistence are driven by T cell survival and homeostatic proliferation. Nat. Med. *15*, 893–900.

Collins, F. (2014). Not sterile after all: the placenta;s microbiome (NIH Director’s Blog).

Eberl, G., Colonna, M., Di Santo, J.P., and McKenzie, A.N. (2015). Innate lymphoid cells. Innate lymphoid cells: a new paradigm in immunology. Science *348*, aaa6566.

CellPress
OPEN ACCESS

Ebrahimi, D., Anwar, F., and Davenport, M.P. (2012). APOBEC3G and APOBEC3F rarely co-mutate the same HIV genome. Retrovirology *9*, 113.

Edgar, R.C. (2004). MUSCLE: multiple sequence alignment with high accuracy and high throughput. Nucleic Acids Res. *32*, 1792–1797.

Einkauf, K.B., Lee, G.Q., Gao, C., Sharaf, R., Sun, X., Hua, S., Chen, S.M., Jiang, C., Lian, X., Chowdhury, F.Z., et al. (2019). Intact HIV-1 proviruses accumulate at distinct chromosomal positions during prolonged antiretroviral therapy. J. Clin. Invest. *129*, 988–998.

Falcinelli, S.D., Kilpatrick, K.W., Read, J., Murtagh, R., Allard, B., Ghofrani, S., Kirchherr, J., James, K.S., Stuelke, E., Baker, C., et al. (2020). Longitudinal dynamics of intact HIV proviral DNA and outgrowth virus frequencies in a cohort of ART-treated individuals. J. Infect. Dis. *224*, 92–100.

Frange, P., Faye, A., Avettand-Fenoel, V., Bellaton, E., Descamps, D., Angin, M., David, A., Caillat-Zucman, S., Peytavin, G., Dollfus, C., et al. (2016). HIV-1 virological remission lasting more than 12 years after interruption of early antiretroviral therapy in a perinatally infected teenager enrolled in the French ANRS EPF-CO10 paediatric cohort: a case report. Lancet HIV *3*, 49–54.

Gandhi, R.T., Cyktor, J.C., Bosch, R.J., Mar, H., Laird, G.M., Martin, A., Collier, A.C., Riddler, S.A., Macatangay, B.J., Rinaldo, C.R., et al. (2020). Selective decay of intact HIV-1 proviral DNA on antiretroviral therapy. J. Infect. Dis. *223*, 225–233.

Garcia-Broncano, P., Maddali, S., Einkauf, K.B., Jiang, C., Gao, C., Chevalier, J., Chowdhury, F.Z., Maswabi, K., Ajibola, G., Moyo, S., et al. (2019). Early antiretroviral therapy in neonates with HIV-1 infection restricts viral reservoir size and induces a distinct innate immune profile. Sci. Transl. Med. *11*, eaax7350.

Gompels, U.A., Larke, N., Sanz-Ramos, M., Bates, M., Musonda, K., Manno, D., Siame, J., Monze, M., Filteau, S., and Group, C.S. (2012). Human cytomegalovirus infant infection adversely affects growth and development in maternally HIV-exposed and unexposed infants in Zambia. Clin. Infect. Dis. *54*, 434–442.

Hartana, C.A., Rassadkina, Y., Gao, C., Martin-Gayo, E., Walker, B.D., Lichterfeld, M., and Yu, X.G. (2021). Long noncoding RNA MIR4435-2HG enhances metabolic function of myeloid dendritic cells from HIV-1 elite controllers. J. Clin. Invest. *131*, e146136.

Hiener, B., Horsburgh, B.A., Eden, J.S., Barton, K., Schlub, T.E., Lee, E., von Stockenstrom, S., Odevall, L., Milush, J.M., Liegler, T., et al. (2017). Identification of genetically intact HIV-1 proviruses in specific CD4(+) T cells from effectively treated participants. Cell Rep. *21*, 813–822.

Houchins, J.P., Lanier, L.L., Niemi, E.C., Phillips, J.H., and Ryan, J.C. (1997). Natural killer cell cytolytic activity is inhibited by NKG2-A and activated by NKG2-C. J. Immunol. *158*, 3603–3609.

Hsiao, N.Y., Zampoli, M., Morrow, B., Zar, H.J., and Hardie, D. (2013). Cytomegalovirus viraemia in HIV exposed and infected infants: prevalence and clinical utility for diagnosing CMV pneumonia. J. Clin. Virol. *58*, 74–78.

Ivarsson, M.A., Loh, L., Marquardt, N., Kekalainen, E., Berglin, L., Bjorkstrom, N.K., Westgren, M., Nixon, D.F., and Michaelsson, J. (2013). Differentiation and functional regulation of human fetal NK cells. J. Clin. Invest. *123*, 3889–3901.

Iyun, V., Technau, K.G., Eley, B., Rabie, H., Boulle, A., Fatti, G., Egger, M., Tanser, F., Wood, R., Fairlie, L., et al. (2020). Earlier antiretroviral therapy initiation and decreasing mortality among HIV-infected infants initiating antiretroviral therapy within 3 Months of age in South Africa, 2006-2017. Pediatr. Infect. Dis. J. *39*, 127–133.

Kamiya, T., Seow, S.V., Wong, D., Robinson, M., and Campana, D. (2019). Blocking expression of inhibitory receptor NKG2A overcomes tumor resistance to NK cells. J. Clin. Invest. *129*, 2094–2106.

Kuhn, L., Paximadis, M., Da Costa Dias, B., Shen, Y., Mncube, S., Strehlau, R., Shiau, S., Patel, F., Burke, M., Technau, K.G., et al. (2021). Predictors of cell-associated HIV-1 DNA over one year in very early treated infants. Clin. Infect. Dis. *74*, 1047–1054.

Kuhn, L., Strehlau, R., Shiau, S., Patel, F., Shen, Y., Technau, K.G., Burke, M., Sherman, G., Coovadia, A., Aldrovandi, G.M., et al. (2020). Early antiretroviral treatment of infants to attain HIV remission. EClinicalMedicine *18*, 100241.

Kumar, S., Stecher, G., Li, M., Knyaz, C., and Tamura, K. (2018). Mega X: molecular evolutionary genetics analysis across computing platforms. Mol. Biol. Evol. *35*, 1547–1549.

Lee, G.Q., Orlova-Fink, N., Einkauf, K., Chowdhury, F.Z., Sun, X., Harrington, S., Kuo, H.H., Hua, S., Chen, H.R., Ouyang, Z., et al. (2017). Clonal expansion of genome-intact HIV-1 in functionally polarized Th1 CD4+ T cells. J. Clin. Invest. *127*, 2689–2696.

Lee, G.Q., Reddy, K., Einkauf, K.B., Gounder, K., Chevalier, J.M., Dong, K.L., Walker, B.D., Yu, X.G., Ndung'u, T., and Lichterfeld, M. (2019). HIV-1 DNA sequence diversity and evolution during acute subtype C infection. Nat. Commun. *10*, 2737.

Lengauer, T., Sander, O., Sierra, S., Thielen, A., and Kaiser, R. (2007). Bioinformatics prediction of HIV coreceptor usage. Nat. Biotechnol. *25*, 1407–1410.

Liu, R., Yeh, Y.J., Varabyou, A., Collora, J.A., Sherrill-Mix, S., Talbot, C.C., Jr., Mehta, S., Albrecht, K., Hao, H., Zhang, H., et al. (2020). Single-cell transcriptional landscapes reveal HIV-1-driven aberrant host gene transcription as a potential therapeutic target. Sci. Transl. Med. *12*, eaaz0802.

Lopez-Verges, S., Milush, J.M., Pandey, S., York, V.A., Arakawa-Hoyt, J., Pircher, H., Norris, P.J., Nixon, D.F., and Lanier, L.L. (2010). CD57 defines a functionally distinct population of mature NK cells in the human CD56dimCD16+ NK-cell subset. Blood *116*, 3865–3874.

Luzuriaga, K., Gay, H., Ziemniak, C., Sanborn, K.B., Somasundaran, M., Rainwater-Lovett, K., Mellors, J.W., Rosenbloom, D., and Persaud, D. (2015). Viremic relapse after HIV-1 remission in a perinatally infected child. N. Engl. J. Med. *372*, 786–788.

Marras, F., Nicco, E., Bozzano, F., Di Biagio, A., Dentone, C., Pontali, E., Boni, S., Setti, M., Orofino, G., Mantia, E., et al. (2013). Natural killer cells in HIV controller patients express an activated effector phenotype and do not upregulate NKp44 on IL-2 stimulation. Proc. Natl. Acad. Sci. USA *110*, 11970–11975.

Martin-Gayo, E., Cole, M.B., Kolb, K.E., Ouyang, Z., Cronin, J., Kazer, S.W., Ordovas-Montanes, J., Lichterfeld, M., Walker, B.D., Yosef, N., et al. (2018). A reproducibility-based computational framework identifies an inducible, enhanced antiviral state in dendritic cells from HIV-1 elite controllers. Genome Biol. *19*, 10.

Maswabi, K., Ajibola, G., Bennett, K., Capparelli, E.V., Jean-Philippe, P., Moyo, S., Mohammed, T., Batlang, O., Sakoi, M., Lockman, S., et al. (2021). Safety and efficacy of starting antiretroviral therapy in the first week of life. Clin. Infect. Dis. *72*, 388–393.

McGary, C.S., Deleage, C., Harper, J., Micci, L., Ribeiro, S.P., Paganini, S., Kuri-Cervantes, L., Benne, C., Ryan, E.S., Balderas, R., et al. (2017). CTLA-4(+)PD-1(-) memory CD4(+) T cells critically contribute to viral persistence in antiretroviral therapy-suppressed, SIV-infected rhesus macaques. Immunity *47*, 776–788.e775.

McMahon, J.H., Chang, J., Tennakoon, S., Dantanarayana, A., Solomon, A., Cherry, C., Doherty, R., Cameron, P., and Lewin, S.R. (2017). Post-treatment control in an adult with perinatally acquired HIV following cessation of antiretroviral therapy. AIDS *31*, 1344–1346.

Mendoza, P., Jackson, J.R., Oliveira, T.Y., Gaebler, C., Ramos, V., Caskey, M., Jankovic, M., Nussenzweig, M.C., and Cohn, L.B. (2020). Antigen-responsive CD4+ T cell clones contribute to the HIV-1 latent reservoir. J. Exp. Med. *217*, e20200051.

Merino, A.M., Sabbaj, S., Easlick, J., Goepfert, P., Kaslow, R.A., and Tang, J. (2013). Dimorphic HLA-B signal peptides differentially influence HLA-E- and natural killer cell-mediated cytolysis of HIV-1-infected target cells. Clin. Exp. Immunol. *174*, 414–423.

Mutanga, J.N., Mutembo, S., Ezeamama, A.E., Song, X., Fubisha, R.C., Mutesu-Kapembwa, K., Sialondwe, D., Simuchembu, B., Chinyonga, J., Thuma, P.E., et al. (2019). Long-term survival outcomes of HIV infected children receiving antiretroviral therapy: an observational study from Zambia (2003-2015). BMC Publ. Health *19*, 115.

Nicoll, G., Ni, J., Liu, D., Klenerman, P., Munday, J., Dubock, S., Mattei, M.G., and Crocker, P.R. (1999). Identification and characterization of a novel siglec,

siglec-7, expressed by human natural killer cells and monocytes. J. Biol. Chem. *274*, 34089–34095.

Palmer, C., Bik, E.M., DiGiulio, D.B., Relman, D.A., and Brown, P.O. (2007). Development of the human infant intestinal microbiota. PLoS Biol. *5*, e177.

Peluso, M.J., Bacchetti, P., Ritter, K.D., Beg, S., Lai, J., Martin, J.N., Hunt, P.W., Henrich, T.J., Siliciano, J.D., Siliciano, R.F., et al. (2020). Differential decay of intact and defective proviral DNA in HIV-1-infected individuals on suppressive antiretroviral therapy. JCI Insight *5*, e132997.

Ramsuran, V., Naranbhai, V., Horowitz, A., Qi, Y., Martin, M.P., Yuki, Y., Gao, X., Walker-Sperling, V., Del Prete, G.Q., Schneider, D.K., et al. (2018). Elevated HLA-A expression impairs HIV control through inhibition of NKG2A-expressing cells. Science *359*, 86–90.

Rinaldi, S., Pallikkuth, S., Cameron, M., de Armas, L.R., Cotugno, N., Dinh, V., Pahwa, R., Richardson, B., Saini, S.R., Rocca, S., et al. (2020). Impact of early antiretroviral therapy initiation on HIV-specific CD4 and CD8 T cell function in perinatally infected children. J. Immunol. *204*, 540–549.

Rose, P.P., and Korber, B.T. (2000). Detecting hypermutations in viral sequences with an emphasis on G–> A hypermutation. Bioinformatics *16*, 400–401.

Rudd, B.D. (2020). Neonatal T cells: a reinterpretation. Annu. Rev. Immunol. *38*, 229–247.

Schonland, S.O., Zimmer, J.K., Lopez-Benitez, C.M., Widmann, T., Ramin, K.D., Goronzy, J.J., and Weyand, C.M. (2003). Homeostatic control of T-cell generation in neonates. Blood *102*, 1428–1434.

SenGupta, D., Brinson, C., DeJesus, E., Mills, A., Shalit, P., Guo, S., Cai, Y., Wallin, J.J., Zhang, L., Humeniuk, R., et al. (2021). The TLR7 agonist vesatolimod induced a modest delay in viral rebound in HIV controllers after cessation of antiretroviral therapy. Sci. Transl. Med. *13*, eabg3071.

Shiau, S., Abrams, E.J., Arpadi, S.M., and Kuhn, L. (2018). Early antiretroviral therapy in HIV-infected infants: can it lead to HIV remission? Lancet HIV *5*, e250–e258.

Simon, A.K., Hollander, G.A., and McMichael, A. (2015). Evolution of the immune system in humans from infancy to old age. Proc. Biol. Sci. *282*, 20143085.

Simonetti, F.R., Zhang, H., Soroosh, G.P., Duan, J., Rhodehouse, K., Hill, A.L., Beg, S.A., McCormick, K., Raymond, H.E., Nobles, C.L., et al. (2021). Antigen-driven clonal selection shapes the persistence of HIV-1-infected CD4+ T cells in vivo. J. Clin. Invest. *131*, e145254.

Technau, K.G., Strehlau, R., Patel, F., Shiau, S., Burke, M., Conradie, M., Sorour, G., Sherman, G.G., Coovadia, A., Murnane, P.M., et al. (2018). 12-month outcomes of HIV-infected infants identified at birth at one maternity site in Johannesburg, South Africa: an observational cohort study. Lancet HIV *5*, e706–e714.

UNAIDS (2019). Global HIV & AIDS Statistics - 2019 Fact Sheet. https://wwwunaidsorg/en/resources/fact-sheet.

Van de Perre, P., Goga, A., Ngandu, N., Nagot, N., Moodley, D., King, R., Moles, J.P., Mosqueira, B., Chirinda, W., Scarlatti, G., et al. (2021). Eliminating postnatal HIV transmission in high incidence areas: need for complementary biomedical interventions. Lancet *397*, 1316–1324.

Van Gassen, S., Callebaut, B., Van Helden, M.J., Lambrecht, B.N., Demeester, P., Dhaene, T., and Saeys, Y. (2015). FlowSOM: using self-organizing maps for visualization and interpretation of cytometry data. Cytometry *87*, 636–645.

Vieira, V.A., Adland, E., Malone, D.F.G., Martin, M.P., Groll, A., Ansari, M.A., Garcia-Guerrero, M.C., Puertas, M.C., Muenchhoff, M., Guash, C.F., et al. (2021). An HLA-I signature favouring KIR-educated Natural Killer cells mediates immune control of HIV in children and contrasts with the HLA-B-restricted CD8+ T-cell-mediated immune control in adults. PLoS Pathog. *17*, e1010090.

Villani, A.C., Satija, R., Reynolds, G., Sarkizova, S., Shekhar, K., Fletcher, J., Griesbeck, M., Butler, A., Zheng, S., Lazo, S., et al. (2017). Single-cell RNA-seq reveals new types of human blood dendritic cells, monocytes, and progenitors. Science *356*, eaah4573.

Violari, A., Cotton, M.F., Kuhn, L., Schramm, D.B., Paximadis, M., Loubser, S., Shalekoff, S., Da Costa Dias, B., Otwombe, K., Liberty, A., et al. (2019). A child with perinatal HIV infection and long-term sustained virological control following antiretroviral treatment cessation. Nat. Commun. *10*, 412.

WHO (2010). Antiretroviral therapy for HIV infection in infants and children: towards universal access - recommendations for a public health approach: 2010 revision. http://www.who.int/hiv/pub/paediatric/infants2010/en/index.html.

STAR★METHODS

KEY RESOURCES TABLE

REAGENT or RESOURCE	SOURCE	IDENTIFIER
Antibodies		
Anti-CD94 BB790	BD Biosciences	624296 (clone HP-3D9)
Anti-CD3 BB700	BD Biosciences	566575 (clone SK7)
Anti-CD4 BB700	BD Biosciences	566452 (clone RPAT8)
Anti-CD8 BB700	BD Biosciences	566392 (clone SK3)
Anti-CD19 BB700	BD Biosciences	566396 (SJ25C1)
Anti-CD20 BB700	BD Biosciences	745889 (clone 2H7)
Anti-CD203c BB700	BD Biosciences	745913 (clone NP4D6)
Anti-CD34 BB700	BD Biosciences	742246 (clone 563)
Anti-CD123 BB660	BD Biosciences	624295 (clone 7G3)
Anti-CD141 BB630	BD Biosciences	624294 (clone 1A4)
Anti-NKG2A VioBright FITC	Miltenyi	130-113-568 (clone REA110)
Anti-CD57 PE-Cy7	Biolegend	359624 (clone HNK-1)
Anti-CD11c PE-Cy5.5	Invitrogen	MHCD11C18 (clone BU15)
Anti-CD294 PE-dazzle 594	Biolegend	350126 (clone BM16)
Anti-Siglec7 PE	Biolegend	339204 (clone G-434)
Anti-CD127 APC-Fire750	Biolegend	351350 (clone A019D5)
Anti-CD64 Alexa Fluor 700	Biolegend	305040 (clone 10.1)
Anti-CD158e1/e2 APC	Miltenyi	130-104-485 (clone REA168)
Anti-CD117 BV750	BD Biosciences	745414 (clone 104D2)
Anti-NKp30 BV711	BD Biosciences	563383 (clone P30-15)
Anti-CD161 BV650	BD Biosciences	563864 (clone DX12)
Anti-CD15 Biotin	Biolegend	301914 (clone HI98)
Anti-CD33 Biotin	Biolegend	303426 (clone W1453)
Streptavidin BV570	Biolegend	405227
Anti-CD16 BV480	BD Biosciences	566108 (clone 3G18)
Anti-NKG2C BV421	BD Biosciences	748169 (clone 134591)
Anti-CD14 BUV805	BD Biosciences	612902 (clone 35E2)
Anti-CD56 BUV737	BD Biosciences	564447 (clone NCAM16.2)
Anti-HLA-DR BUV661	BD Biosciences	565073 (clone G46-6)
Anti-CD86 BUV563	BD Biosciences	741386 (clone 2331FUN11)
Anti-CD1c BUV395	BD Biosciences	742751 (clone F10/21A3)
Biological samples		
PBMC samples from study participants living with HIV-1	Botswana Harvard AIDS Institute Partnership	bhp.org.bw
Chemicals, peptides, and recombinant proteins		
Invitrogen dNTP mix (10mM each)	ThermoFisher Scientific	18427088
AMPure XP beads	Beckman Coulter	A63882
LIVE/DEAD^{TM} Fixable Blue Dead Cell Stain Kit	Thermo Fisher	L23105
Paraformaldehyde solution 4% in PBS	Affymetrix	4243418
FcR Blocking Reagent, human	Miltenyi	130-059-901
Critical commercial assays		
DNeasy Blood and Tissue Kit	Qiagen	69504

(*Continued on next page*)

Continued		
REAGENT or RESOURCE	SOURCE	IDENTIFIER
ddPCR Supermix for Probes (No dUTP)	Bio-Rad	1863024
Invitrogen Platinum Taq DNA Polymerase High Fidelity	ThermoFisher Scientific	11304102
Deposited data		
GenBank	GenBank	accession numbers MK457765 to MK458272 and MZ766582-MZ766922
CTL epitopes (restricted by autologous HLA class I alleles) that match the clade C and B consensus sequence, respectively and CTL escape variants restricted by selected HLA class I alleles and supertypes.	LANL HIV Immunology Database	https://www.hiv.lanl.gov/content/index
The resistance of proviral species to bnAbs.	Bricault et al. (2019)	Bricault et al. (2019)
Viral tropism.	geno2pheno (g2p)	http://coreceptor.geno2pheno.org/
Diagnostic ratios of APOBEC3G or -3F footprints.	Ebrahimi et al. (2012)	Ebrahimi et al. (2012)
Oligonucleotides		
FLIP-Seq oligonucleotides	Millipore Sigma/IDT/Qiagen	N/A
Software and algorithms		
Ultracycler v1.0	Seed and Wang, personal communication	https://dnacore.mgh.harvard.edu/new-cgi-bin/site/pages/viral_genome_sequencing_pages/viral_genome_sequencing_data.jsp
Automated in-house proviral intactness bioinformatic pipeline in Python	Lee et al. (2017)	https://github.com/BWH-Lichterfeld-Lab/Intactness-Pipeline
Los Alamos National Laboratory (LANL) HIV Sequence Database Hypermut 2.0	Rose and Korber (2000)	https://www.hiv.lanl.gov/content/sequence/HYPERMUT/background.html
MUSCLE	Edgar (2004)	http://www.drive5.com/muscle/
MEGA X	Kumar et al. (2018)	version 10.2.2
FlowJo	Tree Star, LLC	version 10.5.3
GraphPad	Prism	version 8.0.1
Other		
QX200 Droplet Digital PCR System	Bio-Rad	https://www.bio-rad.com/en-us/life-science/digital-pcr/qx200-droplet-digital-pcr-system
C1000 Touch Thermal Cycler with 96-Well Fast Reaction Module	Bio-Rad	1851196
DynaMag-96 Side Skirted Magnet	ThermoFisher Scientific	12027
Illumina MiSeq performed by MGH CCIB DNA Core facility	Illumina/MGH CCIB DNA Core	https://dnacore.mgh.harvard.edu/new-cgi-bin/site/pages/index.jsp
Biorender	https://biorender.com	N/A

RESOURCE AVAILABILITY

Lead contact

Further information and requests for resources and reagents should be directed to and will be fulfilled by the lead contact, Mathias Lichterfeld (mlichterfeld@partners.org).

Materials availability

This study did not generate new unique reagents.

Data and code availability

- This paper does not report original code.

CellPress
OPEN ACCESS

- Data were deposited to GenBank with the following accession numbers: MK457765 to MK458272 and MZ766582-MZ766922.
- Any additional information required to reanalyze the data reported in this paper is available from the lead contact upon request.

EXPERIMENTAL MODEL AND SUBJECT DETAILS

PBMC samples were collected in Botswana from participants of the Early Infant Treatment Study (NCT02369406). In addition, PBMC samples were collected in Botswana from control children with HIV-1 infection and ART onset later in the first year of life (N = 10 available at analysis) and from 16 HIV-1 negative infants enrolled in a separate research cohort. Study protocols were approved by the Botswana Ministry of Health's Human Research Development Council, the Harvard T. H. Chan School of Public Health, and the Institutional Review Board of the Brigham and Women's Hospital. PBMCs from an additional cohort of ART-treated adults with HIV-1 infection (n = 41) were recruited at the Massachusetts General Hospital and the Brigham and Women's Hospital (both in Boston, MA, USA). Clinical and demographical characteristics of study participants are summarized in Table S1. Cord blood PBMCs (n = 5) from HIV-negative individuals were acquired from the National Cord Blood Program, New York Blood Center. All the samples from each cohort were collected, cryopreserved and used for experimental assays at the same time to minimize batch effects and other experimental artifacts. Written informed consent was documented from all adult study participants; for underage children, written consent was obtained from their legal caregivers in accordance with the Declaration of Helsinki.

METHOD DETAILS

Sample processing

Blood samples from neonates and infants were collected using heel sticks or venipuncture; samples from adults were obtained by venipuncture. Blood samples were subjected to PBMC isolation using standard Ficoll-Paque density gradient centrifugation.

HIV-1 near-full-genome sequencing

Genomic DNA diluted to single HIV-1 genome levels and subjected to HIV-1 near–full-genome amplification using a one-amplicon or five-amplicon approach (Einkauf et al., 2019) with primer sets adjusted to clade C sequences, as described previously (Lee et al., 2019). PCR products were visualized by agarose gel electrophoresis. Amplification products were subjected to Illumina MiSeq sequencing at the Massachusetts General Hospital (MGH) DNA Core facility. Resulting short reads were de novo assembled using Ultracycler v1.0 and aligned to HXB2 to identify large deleterious deletions (<8000 bp of the amplicon aligned to HXB2), out-of-frame indels, premature/lethal stop codons, internal inversions, or 5′-LTR defect ($\geq$15 bp insertions and/or deletions relative to HXB2), using an automated in-house pipeline written in Python scripting language (https://github.com/BWH-Lichterfeld-Lab/Intactness-Pipeline). Presence/absence of APOBEC3G/3F–associated hypermutations was determined using the Los Alamos HIV Sequence Database Hypermut 2.0 program (Rose and Korber, 2000). Viral sequences that lacked all mutations listed above were classified as ''genome-intact.'' Multiple sequence alignments were performed using MUSCLE (Edgar, 2004). Phylogenetic analyses were conducted using MEGA X, applying maximum likelihood approaches (Kumar et al., 2018). Viral sequences were considered clonal if they had completely identical consensus sequences; single-nucleotide variations in primer binding sites were not considered for clonality analysis. Viral sequences were deposited in GenBank (accession numbers MK457765 to MK458272 and MZ766582-MZ766922). When viral DNA sequences were undetectable, data were reported as LOD, calculated as 0.5 copies per maximum number of cells tested without target identification. Within intact HIV-1 clade C and B sequences, the proportions of optimal CTL epitopes (restricted by autologous HLA class I alleles) that match the clade C and B consensus sequence, respectively and CTL escape variants restricted by selected HLA class I alleles and supertypes described in the LANL HIV Immunology Database (https://www.hiv.lanl.gov/content/index) were determined. The resistance of proviral species to bnAbs were estimated by calculating the number of amino acid signature sites associated with sensitivity to four bnAb classes within the env amino acid sequence from each provirus, as previously described (Bricault et al., 2019). Viral tropism was inferred using geno2pheno (g2p) (http://coreceptor.geno2pheno.org/) (Lengauer et al., 2007). R5 tropism was determined using the Geno to Pheno algorithm with a proportional False Positive Rate (FPR) $\geq$5.75% and non-R5 tropism if FPR <5.75%. Diagnostic ratios of APOBEC3G or -3F footprints, calculated using a previously-described algorithm (Ebrahimi et al., 2012), with a cut-off diagnostic ratio value of >1 and a probability >99%.

Flow cytometry

PBMCs were thawed, stained with LIVE/DEAD Blue Viability Dye (Invitrogen) for 15 min and subsequently preincubated for 10 min with of FcR blocking reagent (Miltenyi). Afterward, cells were incubated for 30 min with different combinations of appropriately titrated antibodies directed against surface and intracellular markers listed in Table S2. Subsequently, the cells were fixed in 2% paraformaldehyde in phosphate-buffered saline (PBS) and acquired on a BD FACSymphony cytometer (BD Bioscience) at the Ragone Institute Imaging Core Facility at MGH. Unstimulated controls were run for each sample and subtracted as background. Data were analyzed using FlowJo v.10.5.3 software (Tree Star LLC) with plugins for T-distributed stochastic neighbor embedding (tSNE) and FlowSOM.

T-SNE analysis was performed in 60,000 cells using equal sampling of cell numbers among timepoints in EIT infants, with 1000 iterations, a perplexity of 5, and learning rate (eta) of 4200 (Van Gassen et al., 2015).

QUANTIFICATION AND STATISTICAL ANALYSIS

Experimental variables between two groups of participants were analyzed using a two-sided Mann-Whitney U test or a Wilcoxon matched-pair rank test, as appropriate. Differences were tested for statistical significance between three or more groups using the two-sided Kruskal-Wallis nonparametric test with post hoc Dunn's multiple comparison test. Statistical associations were assessed using Spearman or Pearson tests. All statistical analyses were performed using GraphPad Prism 8.0.1 and SPICE software.

Cell Reports, Volume *40*

Supplemental information

Immune correlates of HIV-1 reservoir cell decline in early-treated infants

Ciputra Adijaya Hartana, Pilar Garcia-Broncano, Yelizaveta Rassadkina, Xiaodong Lian, Chenyang Jiang, Kevin B. Einkauf, Kenneth Maswabi, Gbolahan Ajibola, Sikhulile Moyo, Terence Mohammed, Comfort Maphorisa, Joseph Makhema, Yuko Yuki, Maureen Martin, Kara Bennett, Patrick Jean-Philippe, Mathias Viard, Michael D. Hughes, Kathleen M. Powis, Mary Carrington, Shahin Lockman, Ce Gao, Xu G. Yu, Daniel R. Kuritzkes, Roger Shapiro, and Mathias Lichterfeld

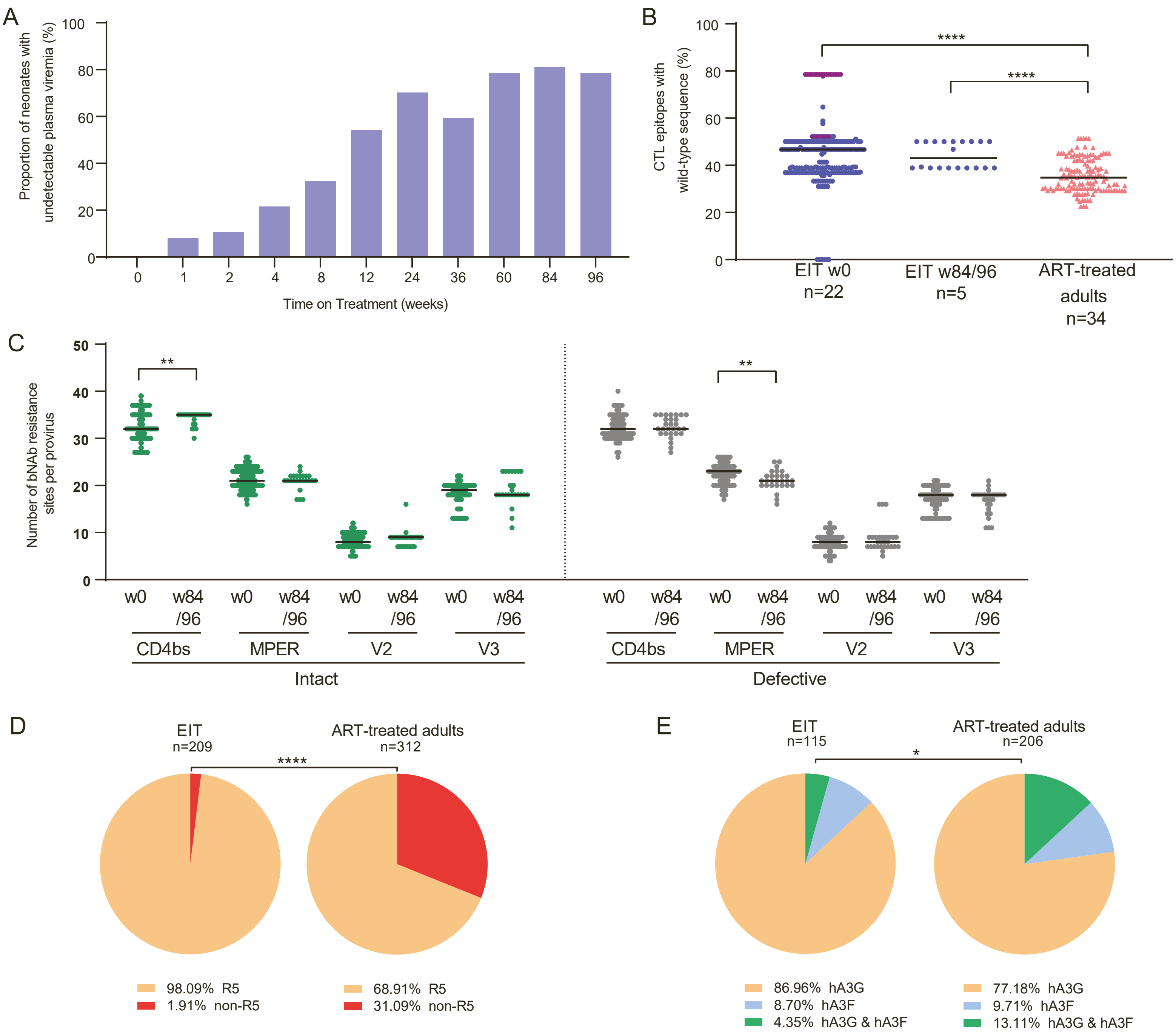

Supplemental Figure 1: Proviral reservoir features in HIV-1-infected neonates. (A): Proportion of neonates in the EIT study with undetectable plasma viremia at indicated timepoints after birth. (B): Proportion of CTL epitopes (restricted by autologous HLA class I alleles) within intact proviruses that display the clade C wild-type sequence (for clade C infected infants from Botswana at week 0 and week 84/96 after early ART) or the clade B wild-type sequences (for clade B-infected adults from the US). ****p<0.0001; Kruskal Wallis test with post-hoc Dunn's test. Data from infants with peripartal infection are indicated in purple. (C): Numbers of amino acid residues associated with resistance to broadly-neutralizing antibodies, determined as described before (Bricault et al., 2019), in intact proviruses from EIT study participants. Data for four classes of bnAbs [CD4 binding site (CD4bs), membrane proximal external region (MPER), V2 domain and V3 domain] at week 0 and week 84/96 are shown. **p<0.01, Mann Whitney U test adjusted for multiple testing. (D): Proportion of R5-tropic and non-R5-tropic intact proviruses from early-treated infants, relative to ART-treated adults. R5 tropism was determined using the Geno to Pheno algorithm with a proportional FPR ≥ 5.75%. ****p<0.0001; Fisher's exact test. (E): Proportion of near-full proviral sequences with footprints of hypermutations mediated by APOBEC3G, APOBEC3F or both. *p<0.05; Chi square test.

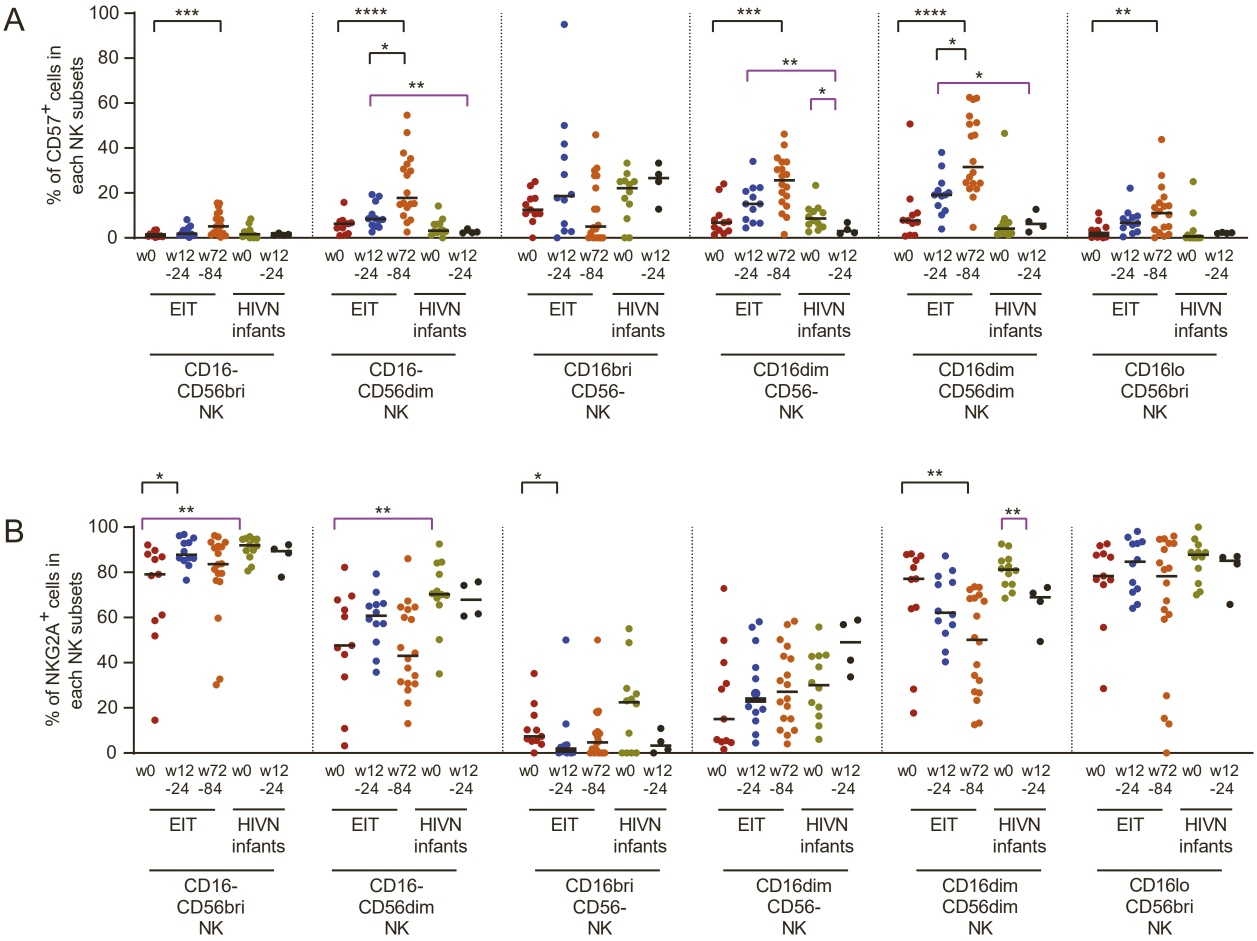

Supplemental Figure 2: NK cell responses of infants receiving early ART compared to HIV-negative infants. (A-B): Longitudinal evolution of CD57-expressing (A) and NKG2A-expressing (B) NK cell subsets in early-treated infants. Data from week 0, week 12/24 and week 72/84 are shown in comparison to HIV-negative infants at week 0 and week 12-24. *p<0.05, **p<0.01, ***p<0.001, ****p<0.0001; Kruskal Wallis test with post-hoc Dunn's test to compare longitudinal data from EIT infants (black line) or Mann Whitney U test adjusted for multiple testing to compare data between similar timepoints from EIT and HIV-negative infants (purple line).

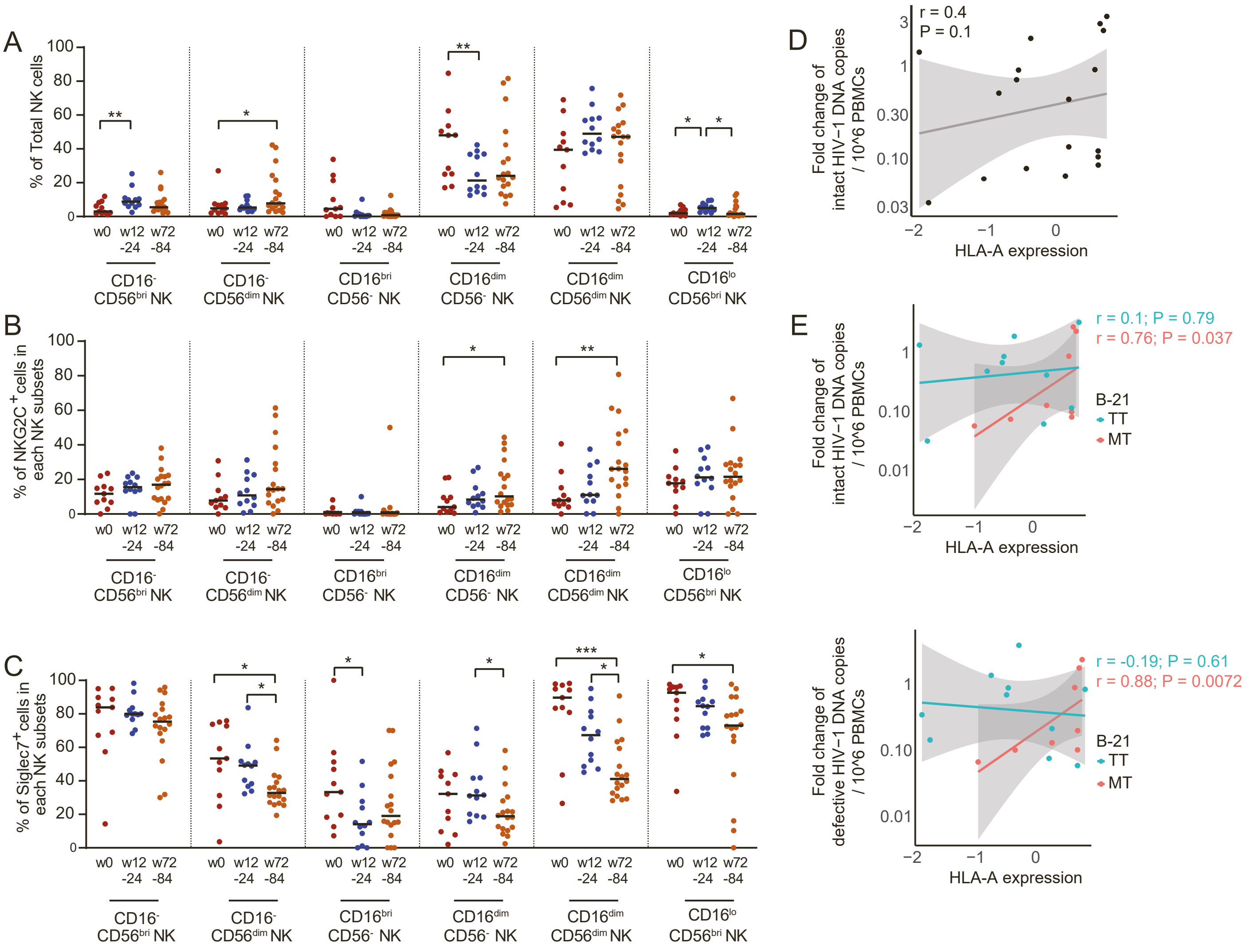

Supplemental Figure 3: Associations between NK cell responses and intact proviral reservoir sequences in early-treated infants. (A): Proportions of indicated NK cell subsets defined by CD16 and CD56 expression in early-treated infants at indicated timepoints. (B-C): Proportions of indicated NK cell subsets expressing NKG2C (B) or Siglec-7 (C) at indicated timepoints. *$p<0.05$, **$p<0.01$, ***$p<0.001$; Kruskal Wallis test with post-hoc Dunn's test. (D): Association between fold-change of intact proviruses between week 0 and week 72/84 and corresponding levels of HLA-A surface expression, determined as described before (Ramsuran et al., 2018). (E): Associations between fold-change of intact (top) and defective (bottom) proviruses between week 0 and week 72/84 and levels of of HLA-A expression. Participants were stratified according to presence or absence of the -21TT or MT genotype. (D-E): Spearman association coefficients are shown.

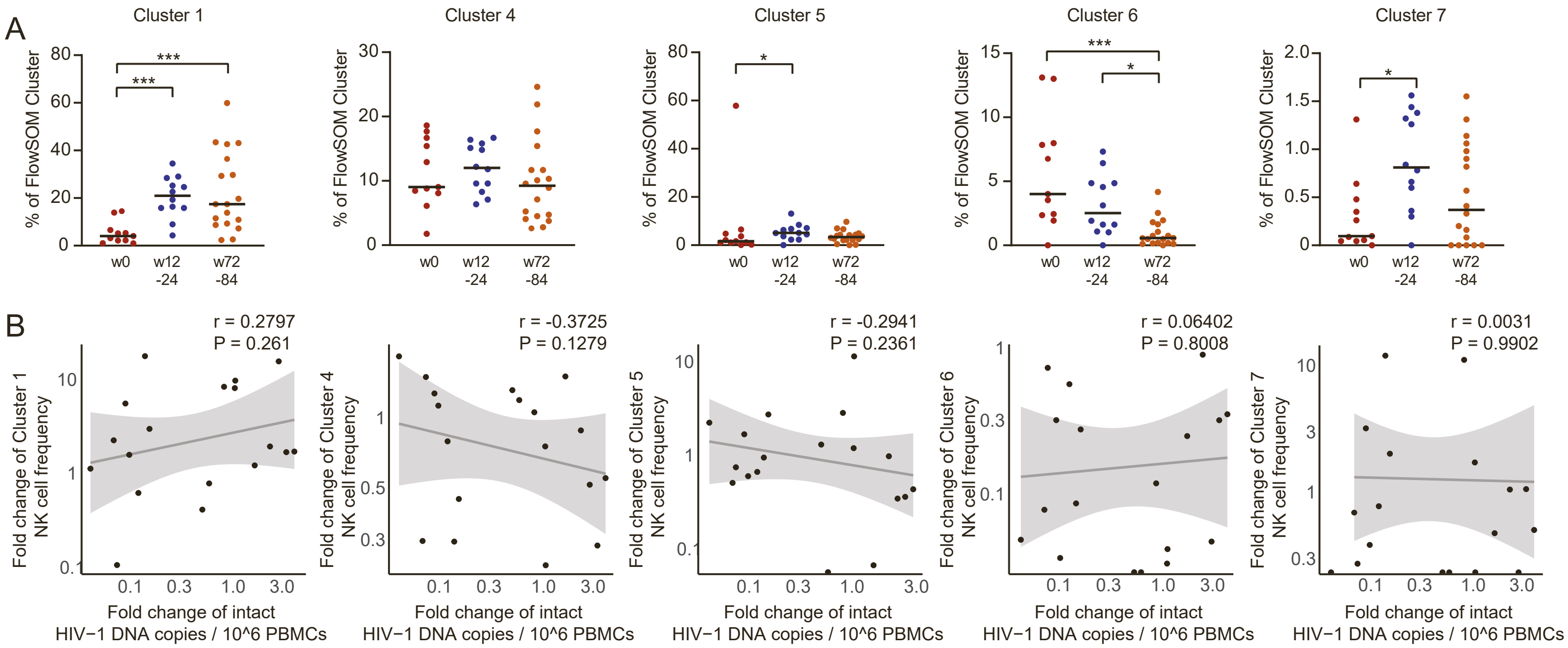

Supplemental Figure 4: Longitudinal associations between NK cell subgroups and intact proviral reservoir sequences in early-treated infants. (A): Longitudinal evolution of NK cell clusters defined through a computational data analysis approach using FlowSOM within the CD56^{dim} CD16^{dim} NK cell compartment, as shown in Figure 3B. (B): Associations between longitudinal changes (between week 0 and week 72/84) of indicated NK cell clusters identified in Figure 3B and corresponding frequencies of intact proviruses. Spearman correlation coefficient is shown. *p<0.05, **p<0.01, ***p<0.001; Kruskal Wallis test with post-hoc Dunn's test.

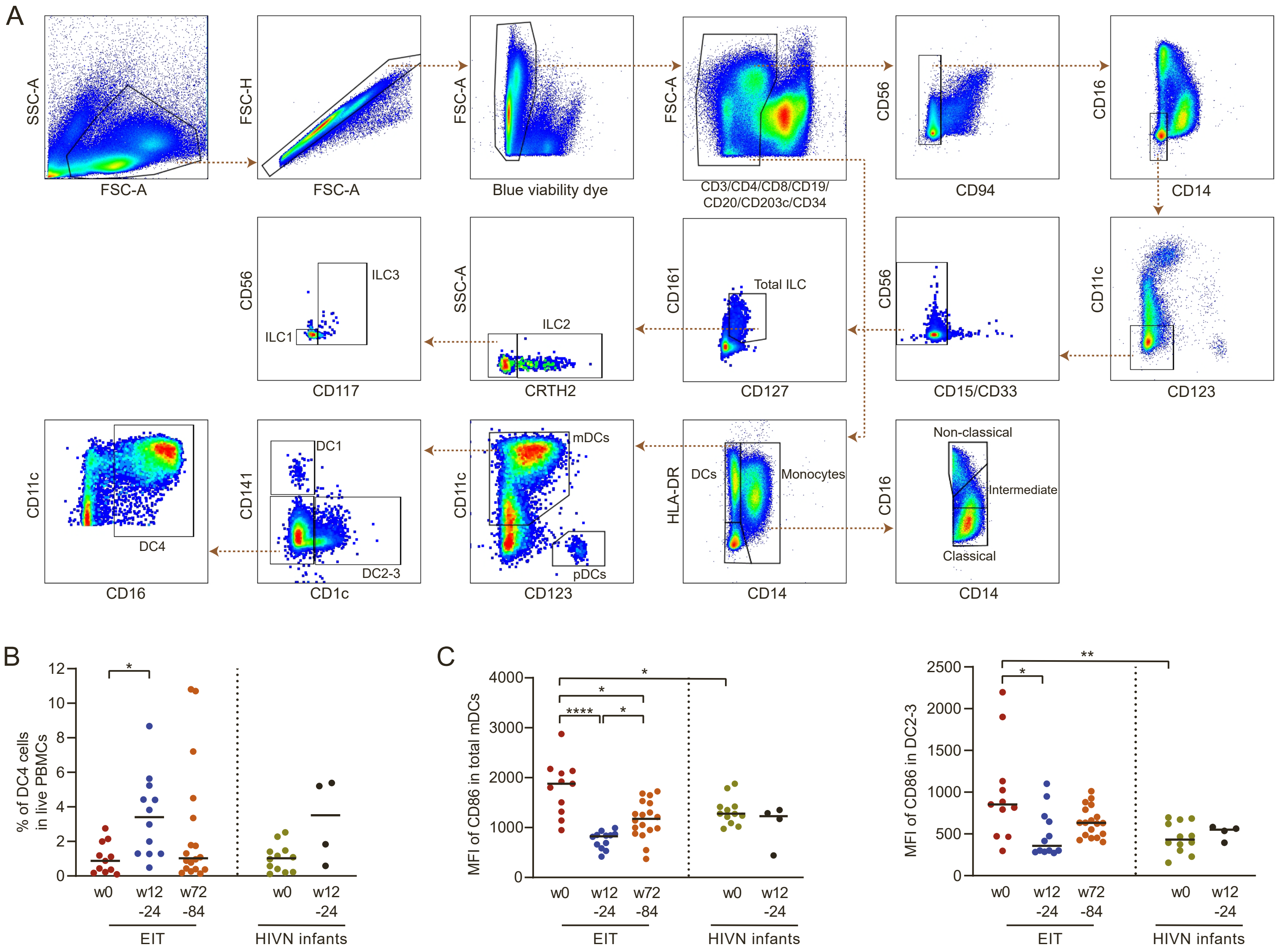

Supplemental Figure 5: Flow cytometric analysis of innate lymphoid cells. (A): Flow cytometric gating strategy for analyzing innate lymphoid cells (ILCs), myeloid dendritic cells (mDCs), plasmacytoid dendritic cells (pDCs) and monocytes. (B): Proportion of DC4 within all PBMC at indicated timepoints in early-treated HIV-1-infected infants (EIT) and in HIV negative infants. (C): MFI of CD86 in total mDCs (left) and DC2-3 (right). *p<0.05, **p<0.01, ****p<0.0001; Kruskal Wallis test with post-hoc Dunn's test and Mann Whitney U test adjusted for multiple testing.

Supplementary Table 1: Clinical and demographical characteristics of study cohort.

Characteristic	EIT n=37	Control infant with later ART initiation n=10	HAART n=41	HIV-1 negative infants n=16
**Age (years)***	As indicated in text	2.2 ± 0.2	53.5 ± 8.6	As indicated in text
**Recruitment site**	Botswana	Botswana	Boston	Botswana
**Time (days) between HIV-1 infection and ART initiation***	mean of 1 day	161.4 ± 95	ND	NA
**Time (years) on ART***	As indicated in text	1.7 ± 0.3	13.1 ± 5.3	NA
**Viral Load (copies/mL)***	887,250.6 ± 2,751,392[#]	<40	<40	NA

*Values are express as mean ± SD

[#] Viral load at enrollment

ND = Not Determined

NA = Not Applicable

Supplementary Table 2: Flow cytometry antibodies used for immune cell phenotyping.

Panel 1: NK/ILC/DC/Monocytes cells					
	**Marker**	**Fluorochrome**	**Clone**	**Reference**	**Vendor**
1	CD94	BB790	HP-3D9	624296	BD Bioscience
2	Lin 1*	BB700	-	*see below	BD Bioscience
3	CD123	BB660	7G3	624295	BD Bioscience
4	CD141	BB630	1A4	624294	BD Bioscience
5	NKG2A	VioBright FITC	REA110	130-113-568	Miltenyi
6	CD57	PE-Cy7	HNK-1	359624	BioLegend
7	CD11c	PE-Cy5.5	BU15	MHCD11C18	Invitrogen
8	CD294 (CHTR2)	PE-dazzle 594	BM16	350126	BioLegend
9	Siglec7	PE	G-434	339204	BioLegend
10	CD127	APC-Fire750	A019D5	351350	BioLegend
11	CD64	AF700	10.1	305040	BioLegend
12	CD158e1/e2	APC	REA168	130-104-485	Miltenyi
13	CD117	BV750	104D2	747514	BD Bioscience
14	NKp30 (CD337)	BV711	P30-15	563383	BD Bioscience
15	CD161	BV650	DX12	563864	BD Bioscience
16	Lin2٢	Biotin	-	٢See below	BioLegend
	streptavidin	BV570	-	405227	BioLegend
17	CD16	BV480	3G18	566108	BD Bioscience
18	NKG2C	BV421	134591	748169	BD Bioscience
19	CD14	BUV805	35E2	612902	BD Bioscience
20	CD56	BUV737	NCAM16.2	564447	BD Bioscience
21	HLADR	BUV661	G46-6	565073	BD Bioscience
22	CD86	BUV563	2331FUN11	741386	BD Bioscience
23	CD1c	BUV395	F10/21A3	742751	BD Bioscience

*CD3/CD4/CD8/CD19/CD20/CD203c/CD34_**566575** (SK7) /**566452** (RPAT8) /**566392** (SK3) /**566396** (SJ25C1) /**745889** (2H7) /**745913** (NP4D6) /**742246** (563)

٢CD15/CD33_**301914** (HI98) /**303426** (W1453)
